# Prevention of mother-to-child transmission of HIV in the MENA region: A systematic review with comparative evidence from Sub-Saharan Africa

**DOI:** 10.1016/j.imj.2026.100240

**Published:** 2026-02-10

**Authors:** SeyedAhmad SeyedAlinaghi, Esmaeil Mehraeen, Sepide Ahmadi, Soudabeh Yarmohammadi, Zohal Parmoon, Amene Abiri, Mahda Malekshahi, Ali Moradi, Soheil Dehghani, Farid Farahani Rad, Zahra Soltanali, Pegah Mirzapour, Shayesteh Jahanfar

**Affiliations:** a Iranian Research Center for HIV/AIDS, Iranian Institute for Reduction of High-Risk Behaviors, Tehran University of Medical Sciences, Tehran 1419733141, Iran; b Research Development Center, Arash Women Hospital, Tehran University of Medical Sciences, Tehran 1653915981, Iran; c Department of Health Information Technology, Khalkhal University of Medical Sciences, Khalkhal 5681761351, Iran; d School of Medicine, Tehran University of Medical Sciences, Tehran 1461884513, Iran; e Trauma Research Center, Kashan University of Medical Sciences, Kashan 8731753153, Iran; f School of Medicine, Ilam University of Medical Sciences, Ilam 6931851147, Iran; g School of Medicine, Public Health and Community Medicine, Tufts University, Boston, MA 02111, USA

**Keywords:** Prevention of mother-to-child transmission, Middle East and North Africa, Sub-Saharan Africa, HIV/AIDS, Mother-to-child transmission

## Abstract

The Middle East and North Africa (MENA) region faces distinct challenges in addressing the HIV epidemic, including social stigma, limited surveillance data, and insufficient coverage of prevention services. Prevention of mother-to-child transmission (PMTCT) of HIV remains a critical public health priority, particularly in regions with rising HIV incidence and constrained health systems. We conducted a systematic review to assess the status of PMTCT in the MENA region, identify gaps in effective implementation, and summarize successful interventions and responses. Due to the scarcity of PMTCT studies originating from the MENA region, high-quality studies from Sub-Saharan Africa were also included to provide comparative and contextual evidence relevant to similar resource-limited settings. Electronic databases (PubMed, Web of Science, and Scopus) were searched for articles published up to March 2024, with no restrictions on study design. Methodological quality and risk of bias were assessed using validated tools. A total of 51 studies were included, comprising data from 32,180 HIV-infected mothers and 171,142 infants. The majority of evidence originated from Sub-Saharan Africa, with limited data available from the MENA region. Antiretroviral therapy during pregnancy, particularly highly active antiretroviral therapy (HAART) and lifelong antiretroviral therapy (Option B +), was consistently associated with substantial reductions in mother-to-child transmission (MTCT) rates. Longer duration of antenatal antiretroviral prophylaxis, appropriate infant antiretroviral regimens, and optimized infant-feeding strategies further contributed to improved outcomes. Non-antiretroviral interventions and maternal genetic factors also influenced transmission dynamics. Despite limited region-specific evidence, this review highlights effective PMTCT strategies that are potentially applicable to the MENA region when informed by comparative evidence from Sub-Saharan Africa. Strengthening access to comprehensive and context-adapted PMTCT services, simplifying treatment pathways, and addressing socio-cultural barriers are essential to reducing vertical HIV transmission in the MENA region.

## Highlights

• PMTCT remains a critical priority in the MENA region.
• Antiretroviral therapy during pregnancy reduces MTCT rates.
• Longer zidovudine prophylaxis lowers placental HIV expression.
• Feeding strategies impact MTCT depending on maternal ART.
• Maternal genetics and non-ART factors influence transmission.

## Graphical abstract

**Figure:**
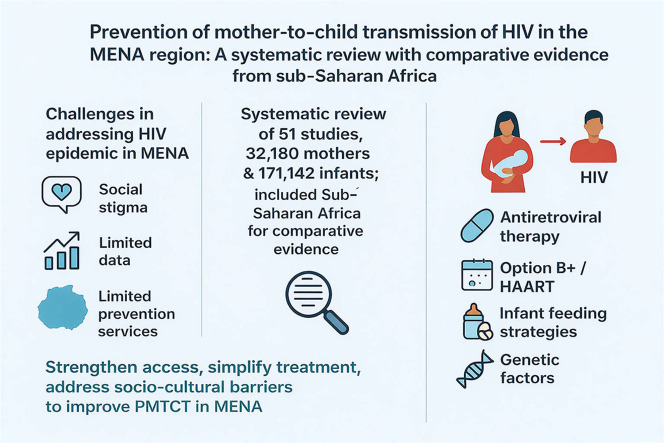

## 1 Introduction

As of 2022, the global human immunodeficiency virus (HIV) epidemic has resulted in approximately 85.6 million infections, with 40.4 million individuals succumbing to acquired immunodeficiency syndrome (AIDS)-related illnesses. The estimated 39 million individuals living with HIV, 1.3 million new infections, and 630,000 AIDS-related deaths in 2022 underscore the persistent global public health challenge posed by HIV.^1^^,^^2^ Addressing this health crisis necessitates a concerted global effort to devise effective treatment and prevention strategies. In response, the Joint United Nations Programme on HIV and AIDS (UNAIDS) has formulated strategies aimed at achieving 95% coverage for HIV testing, access to antiretroviral therapy (ART), and maintenance of viral suppression by 2030,^3^ with the ultimate goal of eradicating the HIV epidemic.^4^

The Middle East and North Africa (MENA) region, encompassing 23 countries, presents a unique set of challenges.^5^ These include social stigma and discrimination against affected populations, suboptimal socioeconomic conditions and healthcare infrastructure, scarcity of HIV data and inadequate surveillance systems, limited resources and funding for HIV programs, particularly in low- and middle-income countries, cultural disparities, and political conflicts and wars.^6^, ^7^, ^8^, ^9^, ^10^ Despite being characterized as a region with low HIV prevalence (< 0.1%), MENA has witnessed the most significant increase in new HIV infections since 2010.^8^^,^^11^ It is one of the few regions globally where HIV infection rates are increasing.^12^ This trend diverges from the UNAIDS above mentioned goals.^13^

Contrary to the global trend of decreasing HIV prevalence, the MENA region exhibits a growing HIV epidemic. This discrepancy was confirmed in the UNAIDS 2022 HIV statistics, which reported a 38% and 51% global reduction in new HIV infections and AIDS-related deaths since 2010, respectively. In contrast, the MENA region has seen a 61% increase in new HIV infections since 2010, although AIDS-related deaths have decreased by 16%.^12^ Furthermore, the projected trends for HIV incidence, mortality, and disability-adjusted life years in the MENA region by 2030 demonstrate a notable upward trajectory.^14^

A critical aspect of the HIV pandemic is vertical transmission, also known as mother-to-child transmission (MTCT), which involves the transmission of HIV from an infected mother to her child during pregnancy, delivery, or breastfeeding. MTCT accounts for over 90% of HIV infections in children. Interventions aimed at preventing pediatric HIV/AIDS and improving the health of mothers and their children, known as “prevention of mother-to-child transmission of HIV” or PMTCT, are a high priority for achieving global elimination targets.^15^^,^^16^ Globally, an estimated 1.3 million HIV-positive women become pregnant each year. Without PMTCT, the rate of HIV transmission from an infected mother to her child ranges from 15% to 45%, with transmission more likely in mothers with high viral loads and/or advanced disease.^16^^,^^17^

UNAIDS data on vertical transmission rates reveal a decrease in global trends from 23% to 11% over the 2010 to 2022 period. However, during the same period, the MENA region only saw a reduction of only 3% points (from 35% to 32%).^12^ The most significant proportion of new child infections is attributed to pregnant women not receiving ART during pregnancy or breastfeeding due to gaps in PMTCT. The coverage of pregnant women receiving antiretroviral (ARV) for PMTCT in the MENA region improved from 9% to 22% between 2010 and 2022. However, when compared to global data, which saw an increase from 48% to 82% coverage, it is evident that the MENA region is lagging in terms of adequate PMTCT services.^12^

Furthermore, the incidence of new HIV infections in children (aged 0–14) in the MENA region has remained relatively constant during the 2010–2022 period, with an estimated 1,700 cases annually. Conversely, the number of infections prevented due to PMTCT in the MENA region is disappointingly low, with estimates ranging from 200 to 500 cases during the same period. In contrast, on a global scale, the number of infections averted due to PMTCT surpasses the number of new HIV infections in children, and this ratio has been progressively increasing over the same timeframe.^12^ Despite the intricacies and challenges, the significance of PMTCT in mitigating the spread of HIV is paramount. Efficacious PMTCT strategies have the potential to safeguard the health of mothers and children, thereby contributing to the objective of eradicating the HIV/AIDS pandemic.

This systematic review is designed to provide a critical examination of the present status of PMTCT, pinpoint deficiencies in effective PMTCT implementation, underscore successful interventions and responses deployed in the region, and propose strategies for enhancement based on empirical evidence. By focusing on the MENA region with comparative evidence from Sub-Saharan Africa, this paper seeks to enrich the understanding of PMTCT, factoring in the region's distinct socio-cultural milieu. The insights gleaned from this review could shape policy formulation, steer the allocation of resources, and ultimately aid in achieving the global ambition of eliminating new HIV infections among children.

## 2 Materials and methods

This systematic review aimed to assess the status of PMTCT in the MENA region, identify deficiencies in effective implementation, and examine successful interventions and responses, while incorporating comparative evidence from Sub-Saharan Africa. This systematic review was registered in PROSPERO (registration number: 1303328).

### 2.1 Data sources

We conducted searches on electronic databases such as PubMed, Web of Sciences, and Scopus up to March 2024. The search strategy was designed to capture all possible keyword combinations, including “HIV” AND “prevention” AND “MTCT” AND “transmission” and their synonym combination.

### 2.2 Inclusion and exclusion criteria

The inclusion criteria required that articles be published in peer-reviewed journals, written in English, and consist of observational studies and randomized controlled trials examining HIV transmission from mother to child in the Middle East and North Africa. Studies were excluded if they were systematic reviews or meta-analyses, if they were duplicate publications and focused exclusively on limited details of HIV transmission without addressing the noted main aim of research such as transmission from mother to child or strategies and lacking experimental data. Due to the scarcity of PMTCT studies originating from the MENA region, high-quality studies from Sub-Saharan Africa were included to provide contextual and comparative insights applicable to similar resource-limited settings, and to inform potential policy and programmatic implications for the MENA region.

### 2.3 Data extraction

Two independent reviewers examined the titles and abstracts to identify potentially eligible systematic reviews and meta-analyses. Full-text screening was conducted to assess the eligibility of the articles based on the specified inclusion and exclusion criteria, resolving any disagreements through discussion and mutual agreement. Data were extracted from the eligible studies using a pre-designed data extraction form, including the first author, study type, country, population, mean age, prevalence, mode of delivery, viral load, number of CD4, strategies to reduce transmission, influence strategies, and findings.

### 2.4 Quality assessment

Validated instruments, including Newcastle-Ottawa Scale and Rob-2 were employed to assess the methodological quality of the studies that were selected (Table 1, Table 2).

**Table 1:** Assessing the risk of bias by Rob-2 tool.

**Table 2:** Bias risk assessment of included studies based on Newcastle-Ottawa Scale.

First author (reference)	Selection (out of 4)	Comparability (out of 2)	Exposure/Outcome (out of 3)	Total (out of 9)
Ameena E. Goga^35^	4	2	3	9
Ameena E. Goga^36^	4	2	3	9
Felicity C. Fitzgerald^37^	3	2	3	8
G. Fatti^38^	3	2	3	8
David Etoori^39^	4	2	3	9
Ali Elgalib^40^	4	1	3	8
Ekouevi Didier K.^41^	3	2	3	8
François Dabis^42^	4	2	3	9
Coutsoudis^43^	4	2	3	9
Carla J. Chibwesha^44^	4	2	3	9
Bulterys^45^	4	2	3	9
Francesca Bisio^46^	4	2	3	9
Lertlakana Bhoopat^47^	4	0	3	7
Dunstan Achwoka^48^	4	0	3	7
Torpey^49^	4	2	3	9
Tookey^50^	4	2	3	9
Tonwe-gold^51^	4	2	3	9
Pellowski^52^	4	2	3	9
Palombi Leonardo^53^	3	1	2	6
Padua E.^54^	3	2	2	7
Naidoo Keshena^55^	4	2	3	9
Muyunda Brian^56^	4	2	3	9
Meda^57^	3	1	3	7
Surasak Taneepanichskul^58^	3	1	3	7
Martinson^59^	3	1	3	7
Lussiana^60^	4	2	3	9
Luo Ma^61^	3	2	3	8
Linguissi^62^	3	2	3	8
V. Leroy^63^	3	2	3	8
Seni Kouanda^64^	3	2	3	8
C. Kilewo^65^	3	2	3	8
D. Ilboudo J^66^	3	2	3	8
J. Ikechebelu J^67^	3	2	3	8
R. M. Hoffman V^68^	3	2	3	8

## 3 Results

### 3.1 Literature Search and Study Selection Flow

**Fig. 1:**
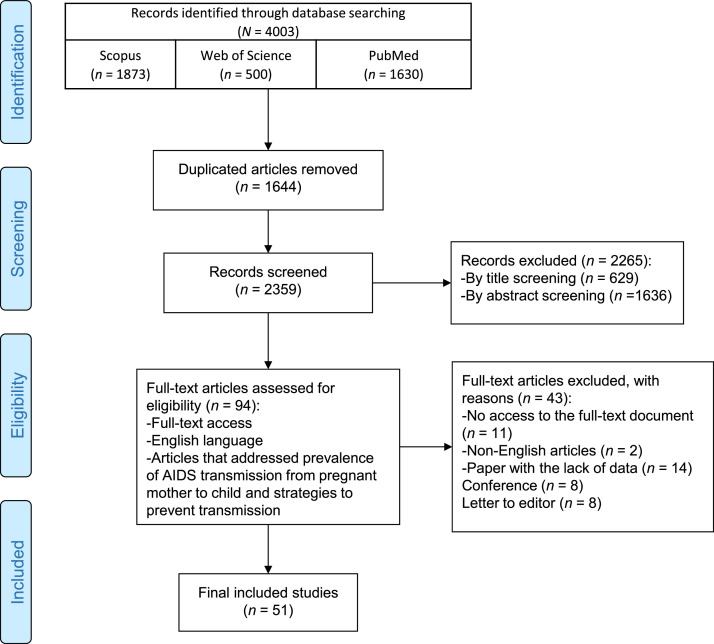
PRISMA 2020 flow diagram of the study retrieval process.

Fig. 1 illustrates the PRISMA flow chart of the literature search process. Initially, a total of (*n* = 4,003) articles were identified. After removing duplicates, (*n* = 1,644) articles were selected for full-text screening. Following this screening process, 51 studies were included in this systematic review. Supplementary material summarizes the characteristics and key findings from these studies (Supplementary Table S1).

### 3.2 Study characteristics and distribution

**Fig. 2:**
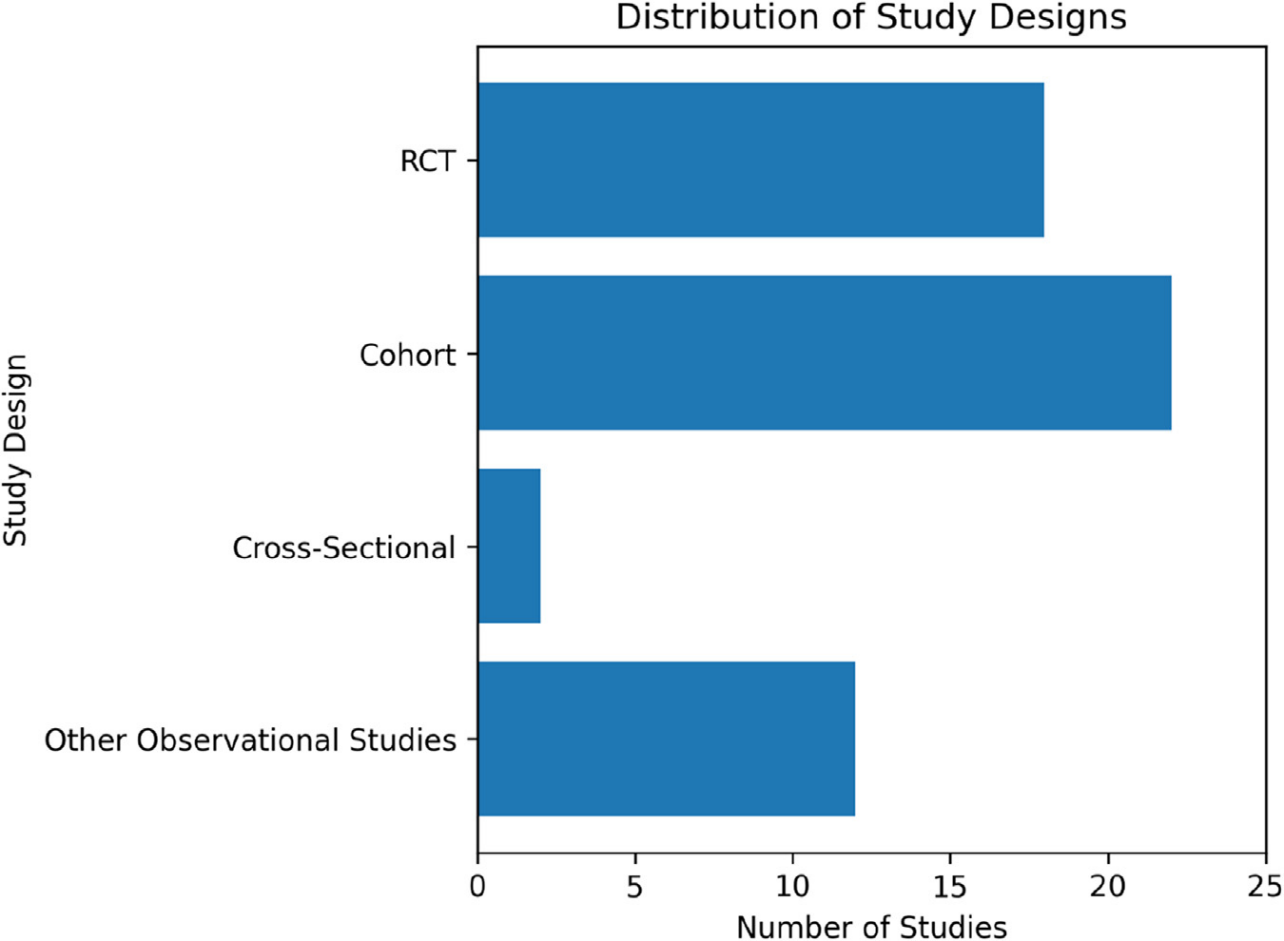
Distribution of the study types.

The review includes a range of study designs: 17 randomized controlled trials (RCTs),^18^, ^19^, ^20^, ^21^, ^22^, ^23^, ^24^, ^25^, ^26^, ^27^, ^28^, ^29^, ^30^, ^31^, ^32^, ^33^, ^34^ 19 cohort studies,^35^, ^37^, ^38^, ^39^, ^40^, ^41^, ^42^, ^43^, ^44^, ^45^, ^47^, ^57^, ^58^, ^59^, ^61^, ^63^, ^64^, ^65^, ^66^ cross-sectional^36^^,^^48^ studies, and other types of observational studies.^46^, ^49^, ^50^, ^51^, ^52^, ^53^, ^54^, ^55^, ^56^, ^60^, ^62^, ^67^, ^68^ (Fig. 2).

**Fig. 3:**
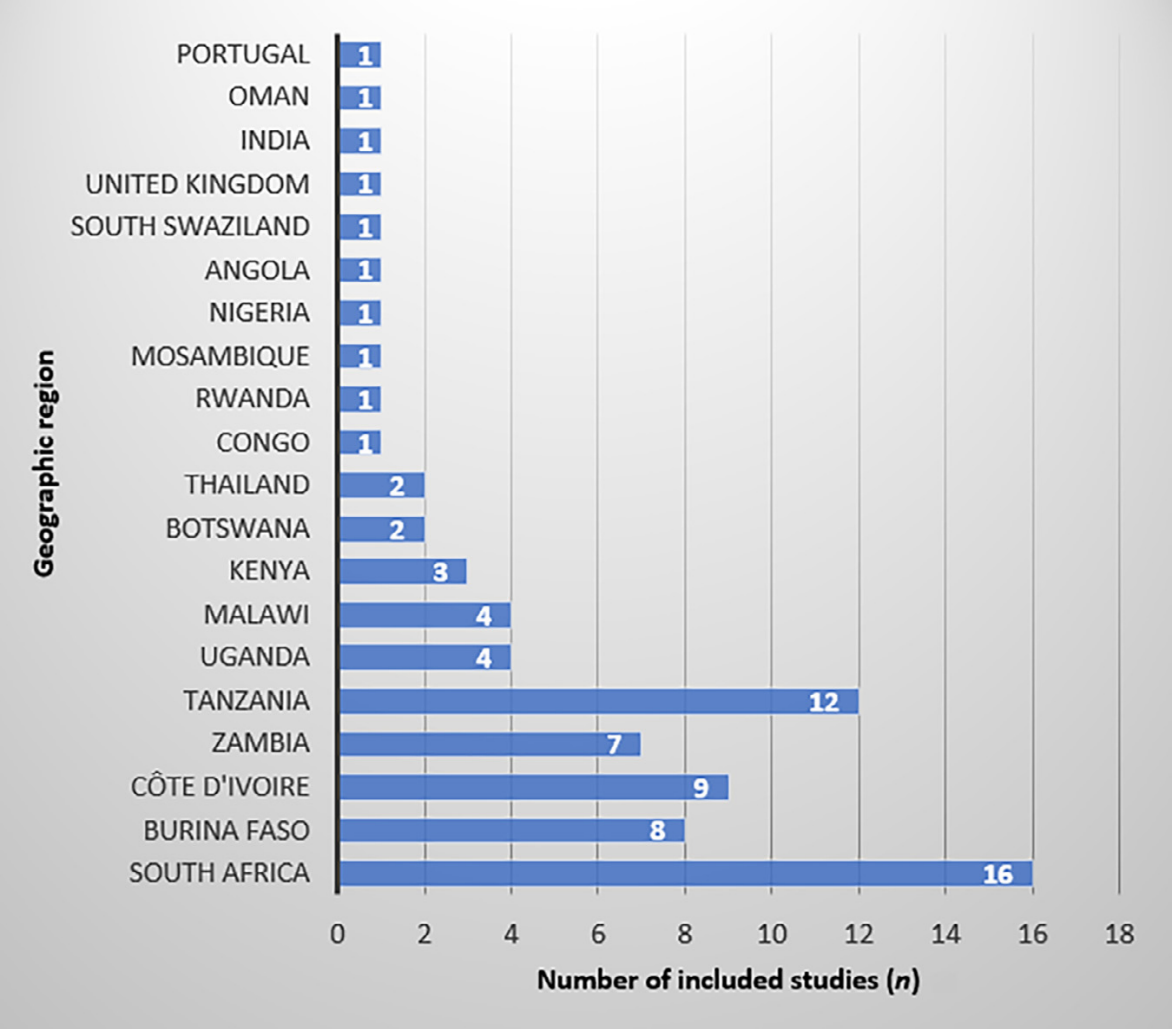
Distribution of the studies in the world.

The geographic distribution of included studies shows a significant focus on Sub-Saharan Africa, with 46 studies conducted across South Africa, East Africa, West Africa, and Central Africa. This regional emphasis highlights the high burden of HIV in Sub-Saharan Africa and the critical need for research in this area. Conversely, research outside Africa is less prevalent, with only one study conducted in India,^20^ and five studies were carried out in other regions, including Oman,^40^ Thailand,^47^^,^^58^ Portugal,^54^ and the UK-Ireland.^50^ Although the primary focus of this review is the MENA region, the geographical distribution of included studies reflects the limited availability of PMTCT research from MENA and the consequent reliance on comparative evidence from Sub-Saharan Africa (Fig. 3).

Studies have assessed the effectiveness of various strategies and factors affecting MTCT rates of HIV. The protocols focus on interventions and factors specific to either mothers or infants. The majority of research compares different antiretroviral drug regimens for mothers, infants, or both. Additional studies investigate the impact of infant feeding protocols, as well as other factors like vaccines, genetic influences in mothers and infants, and the role of maternal sexual health in transmission risk. Collectively, these studies examined a total of 32,180 HIV-infected mothers and 171,142 infants, analyzing various factors, strategies, and outcomes associated with PMTCT of HIV.

### 3.3 Maternal-focused studies

#### 3.3.1 Antiretroviral regimens

ARV therapy during pregnancy was the most commonly studied intervention to reduce MTCT rates. Numerous studies confirm the efficacy of maternal ARV therapy in lowering transmission risk: Goga et al.^36^ reported a significant reduction in early MTCT rates (2.0%) among ARV-treated mothers compared to 10.2% in untreated mothers in South Africa. Similarly, Oman achieved a 1% MTCT rate with 95.5% ARV coverage during pregnancy.^40^ Observational research in South Africa showed a 0.8% MTCT rate with near-universal ARV access,^52^ while Naidoo et al.^55^ found that infants of ARV-treated mothers were 93% less likely to contract HIV despite increased maternal HIV rates during the COVID-19 pandemic.

Taken together, these findings demonstrate consistent benefits of maternal ARV therapy in reducing MTCT, but the variation in transmission rates across studies suggests heterogeneity due to differences in maternal adherence, timing and duration of therapy, and health system capacities.

#### 3.3.2 Single-drug (monotherapy) regimens

Several studies have evaluated antiretroviral monotherapy for reducing MTCT rates. In an RCT, Wiktor et al.^21^ demonstrated that zidovudine (ZDV) (300 mg twice daily from 36 weeks, followed by 300 mg at labor onset and every 3 hours until delivery) significantly lowered MTCT rates compared to placebo. In a 2002 RCT, Leroy et al.^30^ assessed the efficacy of short-term ZDV monotherapy (600 mg from 36 weeks gestation, with an intrapartum dose and continued for 7 days postpartum) over 24 months. The study demonstrated a significant 26% reduction in overall MTCT rates compared to placebo, with efficacy primarily observed in mothers with higher CD4 counts. In a subsequent 2003 RCT, Leroy et al.^29^ focused on the effect of short-term ZDV on postnatal transmission (PT) alone, finding no significant difference between the ZDV and placebo groups. PT rates remained high, especially among women with lower CD4 counts, limiting the long-term efficacy of ZDV. A cohort study by Meda et al.^57^ demonstrated that ZDV monotherapy (300 mg twice daily from 36 weeks, with a 600 mg intrapartum dose and 600 mg daily for 7 days postpartum) was well-tolerated and effective in reducing MTCT rates. Similarly, a cohort study in Thailand^58^ found that a regimen of 250 mg ZDV twice daily from 36 weeks until labor, without additional intrapartum or postpartum doses, was also effective in lowering MTCT rates. A cohort study in 2005^47^ compared the efficacy of long-term ZDV prophylaxis (62 to 92 days; 300 mg twice daily, switching to 300 mg every 3 hours from labor onset until delivery) to short-term ZDV prophylaxis (14 to 35 days before delivery). The results indicated that longer ZDV prophylaxis was more effective in reducing HIV expression in the placenta and lowering MTCT rates. A study compared the safety and efficacy of short-course nevirapine versus zidovudine for HIV-1-infected pregnant women and their infants in Uganda. Nevirapine reduced the risk of HIV-1 transmission by nearly 50% during the first 14–16 weeks of life compared to zidovudine, with transmission rates of 25.1% for zidovudine and 13.1% for nevirapine at this time point. Both regimens were well-tolerated, indicating that nevirapine is a promising, cost-effective option for reducing MTCT in breastfeeding populations.^31^ Another study assessed the efficacy and safety of nevirapine (NVP) versus multiple-dose zidovudine and lamivudine (ZDV/3TC) in PMTCT of HIV-1 during labor in South Africa. HIV-1 infection rates by 8 weeks were 12.3% for NVP and 9.3% for ZDV/3TC (*p* = 0.11). It showed that both regimens are safe, effective and cost-benefit in reducing MTCT rates.^28^ In a study, they found that the effectiveness of single-dose nevirapine (sdNVP) in PMTCT of HIV remained consistent across successive pregnancies, with transmission rates of 11.1% in Soweto for both pregnancies and 13.2% for the first and 5.4% for the second in Abidjan. These results suggest that prior exposure to sdNVP does not impair its efficacy, possibly due to waning viral resistance over time.^59^ Despite overall efficacy, MTCT rates differ across settings, emphasizing the influence of maternal CD4 count, viral load, timing of drug initiation, and breastfeeding practices on outcomes.

#### 3.3.3 Combination regimens

Multiple studies have assessed the efficacy of combination antiretroviral prophylaxis in reducing MTCT rates. An RCT evaluated the addition of lamivudine (3TC) to ZDV across four groups: A (ZDV plus 3TC from 36 weeks gestation, with intrapartum and 7-day postpartum dosing), B (same as A but without the prepartum component), C (intrapartum ZDV and 3TC only), and a placebo group. Results indicated that regimen A was superior to B, B was more effective than C, and C outperformed placebo, highlighting the benefit of longer treatment duration in reducing MTCT.^23^ An observational study by Tookey et al.^50^ found that lopinavir/ritonavir (LPV/r) prophylaxis in pregnant women with HIV effectively reduced MTCT rates from 1.1% (2003–2007) to 0.5% (2008–2012) through successful viral suppression. The regimen was well-tolerated, though a small percentage of infants had congenital abnormalities (2.9%) and low birth weight.

Collectively, these studies highlight that combination regimens, particularly highly active antiretroviral therapy (HAART) and Option B +, achieve the lowest MTCT rates. Heterogeneity in outcomes may be explained by differences in gestational age at therapy initiation, regimen adherence, co-infections, and regional health service coverage.

#### 3.3.4 HAART and long-term ARV regimens

Studies assessing HAART, which uses a combination of three or more ARV drugs, have shown it to be more effective in reducing MTCT compared to simpler regimens. The Kesho Bora Study Group (2011) found that mothers receiving HAART had significantly lower MTCT rates (5.4% at 12 months) compared to those on zidovudine plus single-dose nevirapine (9.5%), especially in breastfeeding populations.^18^ Similarly, Torpey et al.^49^ observed a lower MTCT rate (5.0%) in Zambian infants whose mothers received HAART compared to 20.9% in those without intervention, highlighting HAART's advantage over single-dose regimens. Kouanda et al.^64^ corroborated these findings in Burkina Faso, reporting a 0% MTCT rate at 18 months for mothers on HAART compared to 4.6% for those on short-course antiretroviral therapy (SCART). Ekouevi et al. reported a lower pediatric HIV infection rate with HAART (2.3%) compared to short-course ART (16.1%). However, HAART was associated with a higher incidence of low birth weight.^41^ Another study by Dabis et al.^42^ in Abidjan showed that combining ZDV with sdNVP achieved a 6.5% transmission rate, representing a 72% reduction over ZDV monotherapy. Adding 3TC further reduced transmission to 4.7%, although without significant improvement over ZDV + sdNVP. Padua et al. reported a decline in MTCT rates in Portugal from 7.0% in 1999 to 0.5% in 2005. This reduction was attributed to improved access to HAART and early HIV testing.

In a series of studies examining the effectiveness of different PMTCT interventions, Bisio et al. assessed a prevention program in the Republic of Congo using zidovudine, lamivudine, and nevirapine (ZDV/3TC/NVP) for maternal ARV regimens, alongside single-dose nevirapine at labor onset and infant ZDV for 4 weeks postpartum. Their findings showed a transmission rate of 1.7% among those completing follow-up, though overall estimated transmission in the target group was 10.5%–18%.^46^ Lussiana et al. found that in Angola, maternal ART with ZDV/3TC/NVP during pregnancy reduced infant HIV transmission to 1.5%, compared to 37.1% in those who did not receive ART.^60^ Similarly, Tonwe et al. reported in Côte d'Ivoire that HAART initiated at 24–30 weeks with ZDV/3TC/NVP significantly lowered the peripartum transmission rate to 1.0%.^51^ Linguissi et al. showed that HAART, as opposed to a prophylactic AZT + 3TC + NVP regimen, reduced vertical transmission to 0% among those treated.^62^ Lastly, Ilboudo et al. found that HAART with ZDV/3TC/NVP in HIV/HBV coinfected mothers prevented HIV transmission entirely, though HBV transmission remained at 21.4%.^66^

In Kenya, a cross-sectional study by Achwoka et al.^48^ (2008–2013) evaluated PMTCT interventions among HIV-exposed infants. They found that infants of mothers on combination ART (34.3%, primarily ZDV, 3TC, and NVP) had lower HIV-positivity rates (5.6%) compared to those whose mothers received monotherapy or single-dose NVP (20.4% and 4.5%, respectively). Infant HIV rates varied by feeding type: mixed-fed infants (0–6 months) had higher rates (9.82%) than exclusively breastfed or formula-fed (6.05% and 5.80%). Leroy et al.^63^ reported that short-course ZDV + sdNVP, combined with formula feeding or early breastfeeding cessation (four months), reduced 18-month transmission to 5.6% in Côte d'Ivoire, with reduced transmission even in breastfed infants who stopped early. Finally, Ikechebelu et al^67^ (Nigeria) found a 2.8% transmission rate in non-breastfed infants whose mothers received HAART (ZDV/3TC/NVP) and who received post-exposure prophylaxis, while breastfeeding reduced ART effectiveness, increasing transmission to 12.5%. These findings underscore the importance of early maternal ART, safe feeding practices, and adequate ARV prophylaxis for reducing MTCT, particularly in resource-limited settings. However, MTCT rates varied across studies, reflecting heterogeneity due to differences in study design, maternal adherence, timing and duration of HAART, infant feeding practices, and regional health system capacity. Recognizing these variations is crucial for tailoring PMTCT strategies to specific contexts and populations.

#### 3.3.5 Option *B*+ (lifelong ART)

Studies on lifelong ART, known as Option B + (lifelong ART regardless of CD4 count), have demonstrated its effectiveness in PMTCT. In Swaziland, Etoori et al.^39^ observed a 2.2% transmission rate among pregnant women on Option *B* +, with 91.5% receiving Tenofovir + Lamivudine + Efavirenz and achieving 88.8% viral suppression. In Zambia, Muyunda et al.^56^ reported a 2.9% MTCT rate for Option B +, significantly lower than Option A (CD4-based ART with infant Nevirapine during breastfeeding) and Option B (temporary maternal ART with infant Nevirapine for six weeks).

Studies examining the duration of antenatal HAART reveal that extended treatment before delivery significantly reduces HIV transmission rates. Fitzgerald et al.,^37^ in a cohort, found that among women receiving a median of 7.6 weeks on HAART (Zidovudine, Lamivudine, and Nevirapine), HIV transmission was 5.1%, with no transmission in those treated for over 8 weeks. Chibwesha et al.^44^ reported that Zambian women on HAART for less than 4 weeks before delivery had a 5.2-fold higher risk of transmission compared to those starting at least 13 weeks prior, highlighting 13 weeks as a threshold for optimal PMTCT efficacy. Similarly, Hoffman et al.^68^ observed in Johannesburg that women who became pregnant on HAART had an MTCT rate of only 0.7%, compared to 5.7% for those starting HAART during pregnancy. Each additional week on HAART reduced the risk of MTCT by 8%. No transmissions were observed among women treated for more than 32 weeks before delivery.

In a South African cohort study by Fatti et al., maternal age was found to significantly impact MTCT rates and health outcomes.^38^ Among 956 HIV-positive pregnant women, 32.6% were under 24 years old, and this group exhibited a threefold increase in MTCT risk and reduced antenatal ART uptake. Before April 2010, eligible women received lifelong triple ART if CD4 ≤ 200 cells/µL, while ineligible women received ZDV from 28 weeks and intrapartum sdNVP. After April 2010, eligibility expanded to CD4 ≤ 350 cells/µL, with ZDV starting at 14 weeks for ineligible women. Adolescents faced higher risks of maternal mortality and stillbirth. These findings highlight the need for targeted interventions to improve ART access among young mothers.^38^ Across studies on Option B +, MTCT rates varied depending on maternal age, duration of ART before delivery, adherence levels, and regional healthcare capacities. This heterogeneity emphasizes the importance of context-specific strategies when implementing lifelong ART programs for PMTCT.

### 3.4 Non-ARV interventions and genetic factors

Two randomized controlled trials evaluated non-antiretroviral interventions to reduce MTCT rates. The first trial by Taha et al. involved 1,510 HIV-1-infected women who received either a regimen of metronidazole (250 mg) plus erythromycin (250 mg) antenatally and metronidazole (250 mg) plus ampicillin (500 mg) intrapartum, or a placebo. Antibiotic treatment significantly reduced bacterial vaginosis (23.8% vs. 39.7%, *p* < 0.001). However, no significant differences in MTCT rates were observed at birth or at 4–6 weeks.^26^ The second trial by Kuhn et al. investigated the effects of vitamin A and beta-carotene supplementation among infants born to HIV-positive mothers with Mannose-binding lectin (MBL-2) variants. In this study, 225 infants were followed, with 108 mothers receiving daily supplementation of 5000 IU retinyl palmitate and 30 mg beta-carotene starting between 28 and 32 weeks of gestation. The results showed that infants with MBL-2 variants whose mothers received supplementation had a decreased risk of HIV transmission (odds ratio 0.37; 95% CI: 0.15, 0.91), while those in the placebo group had an increased risk (odds ratio 3.09; 95% CI: 1.21, 7.86), indicating a potential gene-environment interaction affecting susceptibility to HIV.^33^ These findings suggest that while non-ARV interventions alone may have limited overall impact on MTCT, genetic factors and gene-environment interactions can significantly influence outcomes. The variability observed between studies highlights the need to consider both maternal-infant genetics and regional healthcare context when designing PMTCT strategies beyond antiretroviral therapy.

### 3.5 Genetic studies

Besides the RCT done by Kuhn et al.,^33^ Luo Ma et al. conducted a study in Kenya examining the impact of HLA class II genes on MTCT of HIV-1 in drug-naive individuals. Human leukocyte antigen–DR beta chain (DRB) concordance between mother and child increased the risk of perinatal HIV transmission threefold. Conversely, DRB discordance and the presence of the DRB3 phenotype in children were protective against MTCT. Additionally, mothers with the DPB1×55∶01 genotype had a fivefold higher risk of transmitting HIV-1 to their infants, underscoring the role of genetic factors in HIV transmission.^61^

These findings highlight the substantial influence of maternal and infant genetic variability on MTCT risk. The observed differences between studies suggest that genetic susceptibility can modulate the effectiveness of PMTCT interventions, emphasizing the importance of considering genetic profiles alongside antiretroviral and non-ARV strategies when designing region-specific prevention programs.

### 3.6 Infant-focused studies

#### 3.6.1 Antiretroviral regimens

Several studies have been conducted to evaluate the efficacy of antiretroviral prophylaxis in infants to PMTCT of HIV.^18^, ^19^, ^20^^,^^25^^,^^26^^,^^42^ A randomized controlled trial by Flynn et al. compared maternal ART (mART) with infant nevirapine (iNVP) prophylaxis for MTCT prevention throughout breastfeeding in HIV-positive mothers with CD4 counts ≥ 350 cells/mm³. Among 2,431 mother-infant pairs, the mART regimen (TDF/FTC/LPV) and iNVP (age-specific dosing) demonstrated similar transmission rates of approximately 0.57% and 0.58%, respectively, with both groups achieving high HIV-free survival of over 97% at 24 months, confirming the safety and effectiveness of both strategies.^20^

Studies have demonstrated the efficacy of infant ART in reducing MTCT of HIV in settings where maternal ART access is limited. In a randomized trial by Gray et al., infants born to HIV-positive mothers who had not received antepartum ART were given either a single dose of NVP or six weeks of ZDV postnatally. At 12 weeks, infants in the NVP group had lower transmission rates (7.9%) compared to the ZDV group (13.1%), suggesting NVP as a viable postnatal prophylactic option.^19^ Similarly, a trial by Guay et al. in Uganda found that infants whose mothers received a single dose of NVP at labor onset, followed by a single NVP dose to the infant, showed a nearly 50% reduction in MTCT rates by 14–16 weeks compared to infants in the ZDV arm.^31^

Four other RCTs evaluated infant prophylaxis approaches for PMTCT of HIV across varied settings and regimens. In a 2004 study by Taha et al., infants in Malawi whose mothers received intrapartum NVP were randomized to receive either NVP alone or NVP with ZDV. MTCT rates were comparable between groups, with no added safety concerns from ZDV addition.^25^ Another 2003 RCT by Taha et al. in Malawi showed that NVP combined with ZDV postnatally was significantly more effective, reducing MTCT by 36% compared to NVP alone at 6–8 weeks.^24^ Moodley et al.'s study in South Africa evaluated NVP versus ZDV/3TC and found similar MTCT rates between groups at 8 weeks, confirming the effectiveness and safety of both regimens.^28^ Lastly, a trial by Nagot et al. (2009–2012) compared postnatal prophylaxis with lopinavir-ritonavir versus lamivudine in breastfed infants up to 50 weeks and found very low, equivalent rates of HIV-1 transmission, suggesting both regimens are viable options for prolonged prophylaxis through breastfeeding.^27^

In a Tanzanian cohort study by Kilewo et al. (Mitra study), the effectiveness of extended 3TC prophylaxis for preventing HIV transmission through breastfeeding was assessed among infants of HIV-positive mothers who did not need ART for their health. Mothers received ZDV and 3TC from 36 weeks of gestation through one week postpartum, while infants received ZDV and 3TC for the first week and then continued with 3TC alone throughout breastfeeding (up to six months). Cumulative HIV transmission was 3.8% at six weeks and 4.9% at six months, markedly lower than rates observed in the Petra study (5.4% at six weeks and 11.9% at six months), which provided infants ZDV and 3TC for only one week postpartum. This suggests that extended 3TC prophylaxis in infants could effectively reduce breast milk HIV transmission.^65^ In contrast, a randomized controlled trial by Kintu et al. tested the safety and feasibility of the ALVAC—HIV vCP1521 vaccine (administered at birth and 4, 8, and 12 weeks) in HIV-exposed infants in Uganda. The vaccine was well tolerated and did not impact immune responses to routine vaccinations, with no severe adverse events reported, demonstrating feasibility for infant HIV vaccine trials in resource-limited settings. Although not assessing ARV drugs, the vaccine trial further suggests alternative HIV prevention strategies for breastfeeding infants.^32^ Overall, infant ARV prophylaxis is effective, but variations in study results underscore heterogeneity due to timing of maternal ART, infant feeding practices, and duration of prophylaxis.

#### 3.6.2 Infant-feeding protocols

In some studies investigating MTCT of HIV, they had evaluated various infant feeding strategies, yielding insights into HIV-free survival and transmission risks. In a cohort by Goga et al.,^35^ exclusive breastfeeding until 3 months showed limited association with reduced HIV-free survival, particularly in high infant mortality regions where partial breastfeeding increased postnatal infection or mortality risks. Coutsoudis et al.^43^ found that exclusive breastfeeding carried a lower transmission risk than mixed feeding, with comparable rates to formula feeding, suggesting exclusive breastfeeding as a culturally suitable method to limit MTCT in resource-limited settings. The DREAM cohort study by Palombi et al.^53^ demonstrated that breastfeeding alongside HAART was as safe as formula feeding, with similarly low transmission rates (2.2% vs. 2.7% at 6 months). Lastly, a randomized controlled trial by Thior et al.^22^ reported that while formula feeding had a slightly lower MTCT rate than breastfeeding with zidovudine prophylaxis (5.6% vs. 9.0% at 7 months), breastfeeding showed lower mortality rates by 7 months, highlighting both strategies' comparable efficacy in HIV-free survival by 18 months. Collectively, these studies underline that feeding strategies impact MTCT outcomes, but factors like maternal HAART, local resources, and infant mortality influence optimal choices for HIV-positive mothers in different settings.

These findings suggest that while exclusive breastfeeding with maternal ART is generally safe, the impact of feeding strategy on MTCT is context-dependent, reflecting heterogeneity in infant mortality, local resources, and maternal adherence to ART.

This review reveals a diverse range of ARV regimens, with HAART and Option B + showing the strongest evidence for reducing MTCT. Maternal genetic factors targeted non-ARV interventions, and feeding strategies also play a critical role in transmission dynamics. Collectively, these findings underscore the need for integrated, region-specific PMTCT protocols to further reduce HIV transmission rates, particularly in high-burden regions like Sub-Saharan Africa.

## 4 Discussion

### 4.1 Study design diversity

In this systematic review, a broad range of study designs are encompassed, including RCTs, observational studies, and cross-sectional studies. This diversity improves the validation of the results and provides a higher chance of comprehensive evaluation of interventions and their outcomes. To say more, RCTs provided high-quality evidence on antiretroviral regimens, while observational ones presented insights into real implementation and outcomes in settings with limited resources.

### 4.2 Regional and geographic variations

The dominancy of sub-Saharan Africa in terms of geographical distribution of included studies reflects the disproportionate burden of HIV in this region. Similarly, according to the latest released Global HIV & AIDS statistics by UNAIDS, 62% of all new HIV infections were women and girls of all ages in sub-Saharan Africa, and out of 4,000 weekly infected women in the world, 3,100 were in sub-Saharan Africa.^69^ In addition, the low transmission rates observed in countries where ARV coverage surpasses 95%, like in South Africa, suggesting success in PMTCT programs. By contrast, in other regions such as Asia and South America, the limited available data point to a critical gap in research or intervention efforts. The broader geographic representation and investigation in PMTCT and ARV coverage is obligatory in further studies.

### 4.3 Heterogeneity between studies and regions

The included studies show considerable heterogeneity in terms of study design, population characteristics, interventions, duration of ART, and outcome measures. Differences in maternal and infant ARV regimens, timing and duration of therapy, and infant feeding protocols contribute to variations in MTCT rates across studies. Moreover, the predominance of studies from sub-Saharan Africa may influence observed outcomes, as healthcare infrastructure, HIV prevalence, cultural practices, and program implementation differ from the MENA region. These differences should be considered when interpreting the results and applying lessons to other contexts.

### 4.4 Antiretroviral regimens

The findings of this review emphasize the substantial effectiveness of different antiretroviral therapy regimens for both mothers and infants in declining the rate of mother-to-child transmission of HIV.

### 4.5 Maternal ART

The studies investigate ARV regimens, including monotherapy, combination therapy, HAART and long-term, and Option B +. In general, all strategies of maternal ARV treatment during pregnancy, intrapartum, or postpartum lowered the rate of MTCT of HIV.^36^^,^^40^^,^^52^^,^^55^ Similarly, Bhata et al. claimed in their systematic review and meta-analysis that initiation of universal ART considerably lowered the rate of MTCT, from 10.23% to 7.93%.^70^ Another study in Rwanda showed significant protection towards MTCT in mothers and infants who intake ARV prophylaxis.^71^

It is evident that among all types of regimen, HAART and lifelong ART (Option *B* +) demonstrate the best outcomes for reducing MTCT, with rates as low as 0% in some cohorts.^37^^,^^62^^,^^64^^,^^66^^,^^68^ The included study by Linguissi et al^62^ presented 0% MTCT rate among mothers using HAART. This is aligned with the result of the investigation of the European Collaborative Study, which compared the MTCT rate from 1997 to 2004. The results indicating the increasing maternal use of HAART (from 5% to 92%) had led to a significant decrease in MTCT rate from 2.87% to 0.99%.^72^ Also, HAART use in HIV/HBV co-infected mothers completely prevented the transmission of HIV to their children, while HBV transmission rate remained unchanged.^66^ In their study, King et al^73^ investigated the role of co-infection in MTCT of HIV, and found the increased prevalence of co-infections are associated with the increased risk of MTCT. However, infantile vaccines and ARV against co-infection during pregnancy mitigate the risk of transmission.

The cost-effectiveness analysis of PMTCT strategies conducted by Ishikawa et al^74^ indicated that the transition from Option A in 2010 to Option *B* + in 2013 resulted in 33% reduction in MTCT rates. Likewise, the results of one study in Zambia reported 2.9% lower MTCT rate in maternal intake of Option B +, compared to Option A and Option B.^56^

The majority of studies that focused on maternal use of HAART, compared the combination of ZDV + 3TC + NVP efficacy with ZDV + sdNVP,^18^^,^^48^ SCART,^41^^,^^64^ or no intervention.^49^^,^^51^^,^^54^^,^^60^ Other studies only demonstrated the decreased levels of MTCT with the help of HAART.^42^^,^^46^

In terms of maternal HAART duration, several studies found the superiority of extended prenatal HAART over short-course ARVs. Fitzgerald et al^37^ claimed no transmission after 8 weeks on HAART, Chibwesha et al^44^ suggested the optimal threshold for starting HAART is 13 weeks prior to delivery, and a study in Johannesburg requested that initiating HAART 32 weeks before delivery reduces MTCT rate to 0%.^68^ These findings are in line with previous studies presenting that the MTCT rate declines with increasing duration of HAART during pregnancy.^75^^,^^76^

Despite the effectiveness of HAART and Option B +, the results indicate the continued usage of antiretroviral monotherapy, such as ZDV monotherapy and sdNVP, in resource-limited settings. ZDV monotherapy in all phases (during pregnancy, intrapartum, and postpartum) considerably lowered MTCT rates, particularly in those with higher CD4 counts.^21^^,^^30^^,^^57^^,^^58^ Likewise, in a former cohort study by Briand et al,^77^ women who delivered with viral load ≥ 1000 copies/mL showed a higher rate of overall MTCT rate without intravenous ZDV compared to the women with ZDV intake.

Bhoopat et al.^47^, in a cohort study, presented that the long-term use of ZDV (62–92 days until delivery) has more influence on lowering the MTCT rate than short-term ZDV (14–35 days before delivery). However, in a cross-sectional study, the placentas of HIV-infected women who underwent ART containing ZDV were investigated, and the results showed evidence of mitochondrial DNA depletion, alongside increased levels of oxidative stress and apoptosis, all of which were associated with adverse perinatal outcomes.^78^

While single-dose NVP remains cost-effective and logistically practicable, the higher MTCT rates compared to HAART, as reported in studies from Uganda and Côte d'Ivoire,^63^ underline the necessity for transition to combination regimens where achievable. To say more, several studies focused on the burden of viral resistance in both mothers and infants who intake single-dose NVP.^79^, ^80^, ^81^ Yet, in resource-limited settings, specifically where more complex regimens are not available, the first option for the PMTCT is sdNVP.^82^^,^^83^

Combination regimens have been demonstrated to have more efficacy than monotherapy in reducing MTCT rates. The RCT by Tookey et al. showed that lopinavir/ritonavir (LPV/r)-based prophylaxis effectively lowered transmission rates over time.^50^ More prolonged exposure to combination ART also provides a more significant reduction in MTCT rates.^23^ Relatedly, numerous studies demonstrated a significant reduction in vertical transmission of HIV in combination therapy rather than monotherapy.^83^, ^84^, ^85^

### 4.6 Infant ARV

Numerous studies confirm the efficacy of infant ART in mitigating MTCT of HIV. This is more prevalent and efficient in resource-limited settings and when maternal ART is delayed. The former studies showed the primary mother-and-child treatment in Tanzania for reducing MTCT rates is a single dose NVP for mothers during labour and a single dose of NVP for infants within three days after birth.^86^^,^^87^ The results of included studies showed that mART and iNVP are equally effective,^20^ while iNVP usage in infants resulted in lower MTCT rates compared to iZDV.^19^^,^^31^ The extended prophylaxis also demonstrated reduced transmission through breast milk in settings like Tanzania.^65^ A systematic review by Horvath et al^88^ showed extended (14 weeks) nevirapine, or nevirapine with zidovudine infant prophylaxis, has high efficacy in lowering the chance of transmission, compared to two-dose nevirapine prophylaxis. Combination therapy, as in maternal regimens, is claimed to be more effective, particularly during breastfeeding.^24^ Infant regimes, particularly when combined with maternal regimens, offer more feasible and effective solutions for reducing MTCT rates.^89^ Vaccine trials, like ALVAC—HIV vCP1521, also provide favourable substitute strategies for further HIV prevention efforts.^32^

### 4.7 Non-ARV medications

In one study, Taha et al.^26^ found that receiving antibiotics, both in antenatal and intrapartum phases, showed no significant differences in MTCT rates. Notably, this study was conducted before the introduction of NVP prophylaxis to reduce MTCT rates.

### 4.8 Genetic factors

Kuhn et al.^33^ found a significant effect of maternal use of vitamin A and beta-carotene supplements on decreasing MTCT rates in infants with MBL-2 variants. The other study investigated the effect of HLA class II genes on MTCT. Luo et al^61^ found that maternal-child DRB alignment multiplies the risk by three, while DRB variation between mother and infant and the child's DRB3 phenotype are protective.

Former investigations also provide information about the significance of genes in MTCT rates. Yang et al. analyzed the role of p24*gag* and gp41*env* in HIV subtypes. The results of this study showed a prevalence of MTCT among subtype D compared with subtype A.^90^ In a systematic review of 46 articles, *CCR5* promoter, followed by the *CCR2-64I*, was found to have the most effects on reducing MTCT rates. HLA-1 was the most studied factor and was significantly related to the reduction of MTCT rates.^91^

### 4.9 Infant feeding protocols

Exclusive breastfeeding condenses MTCT risk compared to mixed feeding (breastfeeding plus formula) and is comparable to exclusive formula feeding, especially with maternal HAART.^35^^,^^43^^,^^48^^,^^53^ This is in line with the result of several studies, of which Peltier et al. claimed no significant difference in 9-month HIV-free survival in breastfed and formula-fed children. However, the simultaneous intake of HAART with breastfeeding reduced the rate of MTCT.^92^

While formula feeding slightly reduces the rate of MTCT, Thior et al^22^ demonstrated a higher mortality rate in infants compared to breastfeeding with ZDV prophylaxis. This is also demonstrated by Ogundele et al.^93^ that long-term breastfeeding increases the risk of MTCT, although short-term is associated with higher morbidity and mortality of children.

### 4.10 Recent advances and implementation-focused strategies in PMTCT

Recent evidence from the past decade highlights a shift in PMTCT research from efficacy-focused trials toward implementation-oriented and health-system based strategies. Updated WHO and UNAIDS reports emphasize that while biomedical interventions such as Option B + remain central, programmatic factors including early antenatal booking, retention in care, viral load monitoring, and service integration are critical determinants of sustained MTCT reduction.^94^, ^95^, ^96^ Large observational studies and systematic reviews conducted between 2019 and 2024 demonstrate that gaps along the PMTCT cascade particularly loss to follow-up during the postpartum and breastfeeding periods continue to account for residual vertical transmission even in settings with high ART coverage.^97^^,^^98^ These findings suggest that strengthening continuity of care and integrating PMTCT services within broader maternal and child health platforms are essential for achieving and sustaining elimination targets.

### 4.11 Emerging evidence and future directions toward elimination of MTCT

More recent literature also underscores the growing role of context-adapted and innovative approaches in advancing elimination of mother-to-child transmission (EMTCT) goals. Digital health interventions, including mobile health reminders, electronic tracking systems, and decentralized follow-up strategies, have shown promising effects on improving adherence, early infant diagnosis, and maternal retention in PMTCT programs in low-resource settings.^99^^,^^100^ In parallel, post-COVID program evaluations reveal that health-system resilience, task-shifting, and community-based service delivery can mitigate disruptions to PMTCT care and preserve treatment continuity.^101^ Importantly, recent regional analyses from the Middle East and comparable settings indicate that political commitment, stigma reduction, and surveillance strengthening remain pivotal gaps limiting progress toward EMTCT despite availability of effective ART regimens.^102^ Collectively, this evolving body of evidence supports the need for comprehensive, context-specific PMTCT strategies that extend beyond pharmacological interventions to address structural, behavioral, and system-level barriers.

Our study has several limitations. Some included studies had missing data, which may have influenced certain findings. In addition, the scarcity of PMTCT studies from the MENA region limits the representativeness of the results. The predominance of studies from sub-Saharan Africa also restricts the direct generalizability of the findings to the MENA context. Overall, despite consistent evidence supporting the effectiveness of HAART and Option B + in reducing MTCT, the heterogeneity across regions and study designs highlights the need for context-specific adaptations. Interventions successful in sub-Saharan Africa may require modification to address the socio-cultural, economic, and healthcare system differences in the MENA region. Future research should prioritize studies in the MENA region to fill current gaps and enable more direct comparisons.

## 5 Conclusions

Given the cultures and traditions prevalent in the MENA region, as well as in comparable settings in sub-Saharan Africa, there may be a lack of PMTCT services adequately adapted to the needs of pregnant women. To address these gaps, targeted and actionable strategies are recommended, including: implementing culturally sensitive educational campaigns to reduce HIV-related stigma, strengthening healthcare workforce training in PMTCT services, ensuring reliable supply chains for antiretroviral drugs, expanding community-based HIV testing and counseling, and establishing regional data collection systems to guide and monitor interventions. Sustained national commitment, adequate human and financial resources, and provision of essential interventions remain key to success. Interventions should focus on simplifying and improving access to treatment and related services for pregnant women living with HIV, while also prioritizing those most vulnerable to the epidemic.

## CRediT authorship contribution statement

**SeyedAhmad SeyedAlinaghi:** Conceptualization, Formal analysis, Project administration, Supervision, Writing – review & editing. **Esmaeil Mehraeen:** Project administration, Supervision, Writing – review & editing. **Sepide Ahmadi:** Writing – original draft, Data curation, Formal analysis, Project administration, Supervision. **Soudabeh Yarmohammadi:** Writing – original draft, Data curation, Formal analysis, Project administration, Supervision. **Zohal Parmoon:** Writing – original draft, Data curation, Formal analysis, Project administration, Supervision. **Amene Abiri:** Project administration, Supervision, Writing – review & editing. **Mahda Malekshahi:** Writing – original draft. **Ali Moradi:** Writing – original draft. **Soheil Dehghani:** Writing – original draft, Formal analysis, Project administration, Supervision. **Farid Farahani Rad:** Writing – original draft, Formal analysis, Project administration, Supervision. **Zahra Soltanali:** Writing – original draft, Formal analysis, Project administration, Supervision. **Pegah Mirzapour:** Conceptualization, Formal analysis, Project administration, Supervision, Writing – review & editing. **Shayesteh Jahanfar:** Project administration, Supervision, Writing – review & editing.

## Informed consent

Not applicable.

## Organ donation

Not applicable.

## Ethical statement

Not applicable.

## Data availability statement

All data during this study are included in this published article (and its supplementary information file). The data are available from the corresponding author upon reasonable request.

## Animal treatment

Not applicable.

## Generative AI

Artificial intelligence was used to ensure correct English writing style and structure, as well as Graphical Abstract.

## Funding

This research did not receive any specific grant from funding agencies in the public, commercial, or not-for-profit sectors.

## Declaration of competing interest

The authors declare that there is no conflict of interest regarding the publication of this manuscript.

## Appendix Supplementary materials

Image, application 1
