# Supplementary material for: Prevention of mother-to-child transmission of HIV in the MENA region: A systematic review with comparative evidence from Sub-Saharan Africa
Source: Infect Med (Beijing). 2026 Feb 10;5(1):100240. doi: 10.1016/j.imj.2026.100240 (PMC12962071; doi:10.1016/j.imj.2026.100240)
Supplement: Supplementary file 1 [file mmc1.docx]

**Supplementary Table S1.** Characteristics of Studies Included

| ID | **First author (reference)** | **Study type** | **Country** | **Study population (n)** | **Mean Age ±SD** | **Prevalence**  **of mother-to-child transmission of AIDS during pregnancy/ parturition/ breastfeeding** | **Mode of Delivery** | **Number CD4** | **viral load** | **Strategies**  **to reduce transmission** | **Influence strategies** | **Other findings** |
| --- | --- | --- | --- | --- | --- | --- | --- | --- | --- | --- | --- | --- |
| 1 | de Vincenzi I [18] | randomized controlled trial | Burkina Faso, Kenya, and South Africa | Mother:  1. Triple antiretroviral  (n=412)  2.Zidovudine and single-dose  nevirapine (n=412) | Mother: 27 (24–31) | At 6 weeks:  Triple antiretroviral  (**3・3%**)  Zidovudine and single-dose  nevirapine (**5%**)  at 12 months:  Triple antiretroviral  (**5.4%**)  Zidovudine and single-dose  nevirapine (**9.5%**)  (p=0·029) | Vaginal and caesarean section | 200–500 copies per μL  Triple antiretroviral  (336 (282–408))  Zidovudine and single-dose  nevirapine (339 (267–408)) | Triple antiretroviral  (4·23 (3·66–4·75))  Zidovudine and single-dose  nevirapine (4·21 (3·58–4·74)) | 1.During pregnancy and breastfeeding: **triple**  **antiretroviral treatment** (300 mg  zidovudine, 150 mg lamivudine, and 400 mg lopinavir  plus 100 mg ritonavir twice daily until cessation of  breastfeeding (to a maximum of 6·5 months post-partum)  2.ZDV 300mg BD from 34-36 wk until labor + single dose of ZDV (600 mg ) during labor + single dose of NVP (200mg) during labor | Triple antiretroviral prophylaxis during pregnancy and breastfeeding is safe and reduces the risk of HIV  transmission to infants. | *HIV transmission/death: 10.2% (triple ARV) vs. 16.0% (zidovudine/nevirapine) (p=0.017).  *HIV transmission in breastfeeding intention: 5.6% (triple ARV) vs. 10.7% (zidovudine/nevirapine) (p=0.02).  *All infants received a single dose of NVP (0.6 ml oral  suspension, approximately 2 mg/kg body weight) within 72 h  of birth.  * the trial demonstrated a reduction in MTCT rates in this intermediate HIV group. For women with CD4+ cell counts above 500 cells/mm3, the risks of using a triple ARV regimen were deemed to outweigh the benefits, resulting in the administration of prophylaxis stopping around delivery. |
| 2 | Gray Glenda E [19] | randomized controlled trial | South Africa | Infants of HIV-positive women:  -in the ZDV arm: 533  -in the NVP arm: 518 | Infants just after birth | -At  birth, positive HIV PCR : 7.0% in  the NVP-treated infants and 5.8% in the ZDV-treated  infants (p=0.5)  -Cumulative HIV-1  transmission rates at 6 weeks:  11.9% in the NVP arm and 13.6% in the ZDV arm  (p=0.6)  - Cumulative HIV-1  transmission rates at 12 weeks: 14.3% in the NVP arm and  18.1% in the ZDV  arm (p=0.4)  - In infants  uninfected at birth, the postuterine MTCT probabilities  at week 6 in the NVP and the ZDV groups were 5.3% and  8.2%, respectively. At week 12, the postuterine MTCT  probabilities in the NVP and the ZDV groups were 7.9%  and 13.1%, respectively (p=0.06)). | Vaginal and caesarean section | Median: 467× 10 ^6 cells^/l | Median: s 21 800 copies/ml | a **single** oral dose of **NVP** (10 mg/ml oral  suspension at a dose of 2 mg/kg) within 24 h of delivery compared with ZDV (10 mg/ml at a dose of 4 mg/kg administered 12-  hourly for 6 weeks) when  administered postnatally to infants born to HIV-1-  infected women who had not received antepartum or  intrapartum antiretroviral therapy | Administering a **single postpartum dose of NVP** to infants is a valid intervention for preventing mother-to-child transmission of HIV. | factors associated with infection following birth were ZDV use (  p= 0.032), maternal CD4 cell count < 500 × 10^6^ cells/l (  P = 0.007), maternal viral load > 50 000 copies/ml (  P < 0.0001) and breastfeeding (P =0.006). |
| 3 | Patricia M. Flynn [20] | randomized controlled trial | sub-Saharan Africa and India | 2431 mother-infant pairs  Mothers:1220  Infants: 1211 | Mothers: 26 (23-30) | HIV infection rates were similar in both the mART and iNVP arms, with 0.57% and 0.58% of infants affected, respectively. | N/A | in maternal ART group: 682.5 (555-  870)  In infant NVP prophylaxis group: 691 (550-868) | in maternal ART group: 220 (40-1029)  In infant NVP prophylaxis group: 400 (40-1960) | HIV transmission during breastfeeding (mART or iNVP is administered until 18 months post-delivery or breastfeeding cessation, infant HIV-1 infection, or toxicity, whichever comes first.):  - **mART**: (TDF/FTC/LPV: TDF/FTC fixed dose combination tablets containing 300 mg/200 mg orally daily plus LPV/r fixed dose combination tablets, two tablets of 200 mg/50 mg orally twice daily)  - infant nevirapine prophylaxis (**iNVP**): administered with age-specific dosing guidelines: for infants aged birth to 6 weeks with birth weight ≥ 2500 gm, 15mg; for birth weight between 2000-2499 gm, 10mg; for infants over 6 weeks to 6 months, 20mg; for those over 6 months to 9 months, 30mg; and for infants over 9 months until breastfeeding cessation or 18 months, whichever comes first, 40mg | Both **mART** and **iNVP** prophylaxis strategies demonstrated safety and were linked to minimal breastfeeding HIV-1 transmission and high infant HIV-1-free survival at 24 months. | over 97% HIV-1-free survival at 24 months, despite lower-than-expected transmission rates affecting statistical power. Infant mortality was notably low at 1.7%. |
| 4 | Wiktor [21] | randomized controlled trial | Côte d'Ivoire | 276 women:  Placebo:137  Zidovudine:139 | (IQR =26(22-30)) | Placebo: 26.1%  Zidovurdine:16.5%  among infants at age 3 months | N/A | IQR:  Placebo: 548(350-728)  Zidovurdine: 528(245-707) cells/µl | N/A | **zidovudine** (300 mg tablets) one tab twice a day from 36 week’s gestation until the onset of labour, one tab at the onset of labour and one tab every 3 hour until delivery | The estimated transmission rates in the **zidovudine** group were 44% lower at 4 weeks and 37% lower at 3 months than in the placebo group. | Efficacy did not differ significantly with maternal CD4 count or duration of or adherence to prepartum or intrapartum treatment regimens |
| 5 | Thior Ibou [22] | randomized controlled trial | Botswana | Infants:  Breastfed Plus Zidovudine:598  Formula-Fed:602 | N/A  Median:  Total:26.78  Breastfed Plus Zidovudine:26.87  Formula-Fed:26.76 | 7-month infection rates:5.6% (32 infants in the formula-fed group) vs 9.0% (51 infants in the breastfed plus zidovudine group) (P=.04; 95% CI for difference, –6.4% to –0.4%) | N/A | Total: 366.0  Breastfed Plus Zidovudine: 372.0  Formual-fed: 358.5  Cells/mm3 | Maternal baseline:  4.35Log10 copies/ml | **Zidovudine** 300 mg orally for all mothers twice daily from 34 week’s gestation during labour  Part 2 of the trial to reduce postnatal HIV infection and mortality, for infants breastfed prophylactic infant **zidovudine** for 6 months and for those formula fed 1 month of prophylactic zidovudine. | The 7-month HIV infection rates were 5.6% in the formula-fed group versus 9.0% in the breastfed plus zidovudine group (P = .04; 95% CI for difference, -6.4% to -0.4%).  Breastfeeding with zidovudine prophylaxis was not as effective as formula feeding in preventing postnatal HIV transmission, but was associated with a lower  mortality rate at 7 months. Both strategies had comparable HIV-free survival at 18  months. | Cumulative mortality or HIV infection rates at 18 months were 80 infants (13.9%, formula fed) vs 86 infants (15.1% breastfed plus zidovudine) (P=.60; 95% confidence interval for difference, –5.3% to 2.9%). Cumulative infant mortality at 7 months was significantly higher for the formulafed group than for the breastfed plus zidovudine group (9.3% vs 4.9%; P=.003), but this difference diminished beyond month 7 such that the time-to-mortality distributions through age 18 months were not significantly different (P=.21). |
| 6 | Petra Study Team [23] | randomized controlled trial | Tanzania, south Africa, Uganda | 1457 women:  RegimenA:366  regimenB:371  regimenc:368  placebo:352 | (IQR  regimenA: 26(23-29)  RegimenB:26(23-30)  RegimenC:26(23-30)  Placebo:26(22-30)) | Week 6 HIV-1 transmission rates:  5·7% for regimen A, 8·9% for regimen B, 14·2% for regimen C, and 15·3% for the placebo group | Caesarean section:  regimenA:33%  regimenB:35%  regimenC:32%  placebo:33% | Median:  RegimenA: 445 (295–612)  RegimenB: 475 (300–673)  regimenC: 440 (283–613)  placebo: 435 (290–605) | N/A | four regimens: **A**, **zidovudine** plus **lamivudine** starting at 36 weeks’ gestation, followed by oral intrapartum dosing and by 7 days’ postpartum dosing of mothers and infants; **B**, as regimen A, but without the prepartum component; **C**, intrapartum **zidovudine** and **lamivudine** only, or placebo | Early efficacy at week 6 p (relative risk [95% CI]):  RegimenA: 0·37 (0·21–0·65)  regimenB: 0·58 (0·36–0·94)  regimenc: 0·93 (0·62–1·40)  placebo:1.00 | Following week 6, continued HIV-1 transmission (not taking death into account) was rarely seen among non-breastfed children and was predominantly found in breastfed infants. Due to weaning and HIV transmission, the proportion of breastfed children who were not HIV-1 infected (the children at risk) declined over time from 65% after 3 months of follow-up to 53% after 6 months, 31% after 1 year and 20% after 18 months |
| 7 | Taha 2003 [24] | randomized controlled trial | Malawi | 894 infants:  NVP only:448  NVP+ZDV:446 | Maternal age:  NVP: 24.8 (4.7)  NVP+ZDV: 24.8 (4.7) | *At birth:  NVP only:8.1%  NVP+ZDV:10.1% (p=0.3)  *at 6 to 8 weeks:  NVP only:14.1% (95% CI [CI], 10.7%-17.4%)  NVP + ZDV:  16.3% (95% CI, 12.7%-19.8%)  (p=0.36)  *For infants  not infected at birth and retested at 6 to 8 weeks:  NVP only:6.5%  NVP+ZDV:6.9% (p=0.88) | NVP:  Spontaneous vaginal:94.1  Cesarean:3.5%  Other:2.4%  NVP+ZDV:  Spontaneous vaginal:97.9%  Cesarean:1.1%  Other:1.0% | N/A | Mean (SD) maternal viral load(log10): NVP only  :4.4 (0.77)  NVP + ZDV: 4.4 (0.76) | Mothers received a 200-mg **single oral dose of** **NVP** intrapartum and infants received either 2-mg/kg oral dose of **NVP** or **NVP** (same dose) **plus** 4 mg/kg of **ZDV** twice per day for a week | The frequency of mother-to-child HIV transmission at 6 to 8 weeks in 2 study groups was comparable with that observed for other perinatal HIV intervention studies among breastfeeding women in Africa. The safety of the regimen containing neonatal ZDV was similar to that of a standard NVP regimen.  *no significant difference between the two groups. | a clear trend of increase in mother-to-child transmission with increase in maternal viral level in each of the treatment group |
| 8 | Taha 2004 [25] | randomized controlled trial | Blantyre and Lilongwe, Malawi; Dar es Salaam, Tanzania;Lusaka, Zambia | 2294 women:  Placebo:1146  antibiotics:1146  liveborn babies :1510 | Maternal:  Antibiotics: 25.3 (0.18)  Placebo: 25.2 (0.17) | At birth:  Antibiotics:7.1%  Placebo: 8.3%  at 4–6 weeks:  antibiotics:16.2%  placebo:15.8% | Vaginal delivery (versus cesarean section):  Antibiotics: 95.0% (0.8%)  Placebo: 94.5% (0.8%) | Median maternal (cells/µl):  Antibiotics: 338 (214–495)  Placebo: 335 (205–489) | Mean maternal (log10):  Antibiotics: 4.3 (0.03)  Placebo: 4.3 (0.03) | **Nevirapine** for all HIV-positive women and infants  The Trial: At 20–24 weeks’ gestation,  **metronidazole** 250 mg and **erythromycin** 250 mg by mouth three times a day for 7 days. A **second** oral course of antibiotics consisting of **metronidazole** 250 mg and **ampicillin** 500 mg at the onset of labor every 4 h, continuing after delivery three times a day until the course was completed in the intervention group | no statistically significant differences between two groups at birth or at 4-6 weeks | the proportion of women with bacterial vaginosis at the second antenatal visit was significantly lower in the antibiotics arm compared with the placebo arm (23.8 versus 39.7%; P < 0.001), but the frequency of histological chorioamnionitis was not different (antibiotics 36.9%; placebo 39.7%; P ¼ 0.30). |
| 9 | Taha 2006 [26] | randomized controlled trial | Malawi | 1119 babies:  NVP only:  557  NVP + ZDV:562 | Maternal age:  NVP+ZDV: 25·2 (4·7)  NVP: 24·9 (4·6%) | At birth:  NVP only:10.2%  NVP+ZDV:9.0%  At 6-8 weeks:  NVP only:12.1%  NVP+ZDV:7.7%  overall rate at 6–8 weeks:  NVP+ZDV:15.3%  NVP only:20.9% | Spontaneous vaginal:  NVP+ZDV:99.5%  NVP:99.3% | N/A | Mean maternal(log10):  NVP+ZDV: 4·50 (0·79)  NVP only: 4·55 (0·85%) | **nevirapine** (2 mg/kg weight) as a single dose immediately after birth to all babies and **zidovudine** twice daily for 1 week (4 mg/kg weight) for babies randomized to the NVP+ZDV group | nevirapine and zidovudine compared with nevirapine only: a protective efficacy of 26·8% against infection up to 6–8 weeks and a 36·4% (4·5–52·0) efficacy of NVP+ ZDV compared to NVP only for babies who were HIV negative at birth | lowering maternal viral load (through antiretroviral therapy) coupled with infant prophylaxis with nevirapine and zidovudine could substantially reduce rates of mother-to-child transmission. |
| 10 | Nagot Nicolas [27] | randomised controlled trial | Burkina Faso, South Africa, Uganda and Zambia | 1236 infants:  lopinavir-ritonavir: 615  lamivudine:621 | N/A  (median maternal age:  Lopinavir-ritonavir:: 27·1 (23·8–31·2)  lamivudine: 27·0 (22·9–30·9)) | between age 7 days and 50 weeks of breastfeeding:  lopinavir-ritonavir: 1·4% (95% CI 0·4–2·5)  lamivudine: 1·5% (0·7–2·5) | N/A | lopinavir-ritonavir: 528 (430–667)  lamivudine: 531 (437–673)  pre-delivery: (cells per μL) | Median (log10 copies per mL):  lopinavir-ritonavir: 3·4 (2·9–3·9)  lamivudine: 3·4 (3·0–3·9) | Maternal **PMTC** regimen during pregnancy and labour +randomization of  HIV-1-uninfected breastfed infants aged 7 days to either **lopinavir**–**ritonavir** or **lamivudine** (pediatric liquid formulations, twice a day) up to 1 week after complete cessation of breastfeeding or at the final visit at week 50 | No superiority of lopinavir-ritonavir to lamivudine (hazard ratio [HR] of lopinavir–ritonavir versus lamivudine of 0·90, 95% CI 0·35–2·34; p=0·83).  Both drugs led to very low rates of HIV-1 postnatal transmission for up to 50 weeks of breastfeeding. | N/A |
| 11 | Moodley [28] | randomised controlled trial | South Africa | 1317 women:  NVP:665  Zdv /3TC:662  1329 infants  NVP:663  Zdv /3TC:666 | N/A  (median: 25) | Overall rate through 8 weeks:  NVP: 12.3% (95% CI, 9.7–15.0)  Zdv/3TC :9.3% (95% CI, 7.0–11.6) | Cesarean section: 390 (29.6)  Vaginal: 920 (69.9)  No record: 7 (0.5) | at delivery, cells/mm3:  Mean (SD):  NVP: 436 (246)  Zdv/3TC: 436 (253) | Maternal HIV-1 RNA level at delivery, log10 copies/mL  Geometric mean:  NVP: 10,351  Zdv/3TC: 7674 | **Mothers**:  **NVP**:  200 mg of Nvp orally in labor plus an additional dose 48 h later if still in labor, followed by 200 mg 24–48 h postpartum  **ZDV/3TC**: a loading dose of 600 mg of Zdv and 150 mg of 3TC orally, followed by 300 mg of Zdv every 3 h and 150 mg of 3TC every 12 h until delivery. continueing on a twice-daily dose of Zdv (300 mg) and 3TC (150 mg) for 1 week after delivery  **Infants**:  **NVP**:  6 mg of Nvp oral suspension (10 mg/mL) 24–48 h after delivery  **Zdv/3TC**:  Weighted > 2kg twice-daily doses of Zdv syrup (12 mg) and 3TC oral solution (6 mg) 12 h after birth and continuing for 1 week  weighed <2 kg: Zdv (4 mg/kg) and 3TC (2 mg/kg) | Both regimens were similarly effective:  HIV-1–free survival rates for the Nvp: 85.9% (95% CI, 82.9–88.9)  For Zdv/3TC: 87.5% (95% CI, 84.7–90.3) | Common complications: obstetrical for mothers (Nvp group, 24.3%; Zdv/3TC group, 26.3%) and respiratory for infants (Nvp group, 16.1%; Zdv/3TC group, 17.0%) |
| 12 | Leroy [29] | randomized  double-blind placebo-controlled trials (DITRAME-ANRS049a & RETRO-C) | Abidjan &  CoÃ te d'Ivoire & Bobo-Dioulasso & Burkina-faso | Intervention on mothers.  Zidovudine:  Mothers:349  254 infants  Placebo:  Mothers:352  225 infants | Median age at entry:  Zidovudine:  25 (19–35)  Placebo:  25 (19–36) | Postnatal transmission: At age 24 months  Zidovudine:  9.1%  Placebo:  8.4  (p= 0.87)  Overall: 42 infected children 0f 479 | N/A | Median CD4 count at entry (× 106 cells/l)  Zidovudine:  598 (175–1065)  Placebo:  558 (210–1164) | Median Log10 viral load at entry (copies/µl)  Zidovudine:  3.85 (3.27–4.45)  Placebo:  3.80 (3.36–4.33) | consenting pregnant HIV-1 seropositive women with hemoglobin > 70 g/l were randomized at 36–38 weeks’ gestation to receive oral **zidovudine** (250 mg or 300 mg) or a matching **placebo**: one tablet twice daily until the beginning of labor, then a single oral dose of 500 mg or 600 mg or one 300 mg tablet every 3 h until delivery, then a 7-day postpartum maternal treatment of 500 or 600 mg per day (DITRAME only). No study drug was given to the neonate. | Postnatal transmission occurred at a **similar** rate between two groups and therefore reduced the long-term overall efficacy of this peripartum **zidovudine** regimen at age 24 months | The higher risk of Postnatal transmission among women with low CD4 cell count |
| 13 | ValeÂriane Leroy [30] | randomized  double-blind placebo-controlled trials (DITRAME-ANRS049a & RETRO-C) | Abidjan &  CoÃ te d'Ivoire & Bobo-Dioulasso & Burkina-faso | 1)Mothers Zidovudine:336  2)Mothers Placebo:342  3)Children Zidovudine:319  4)Children Placebo:322 | 1)25 (22-29)  2)25 (22-30) | 1)68 (21.3%)  2)94 (29.2) | NA | 1)545(355-732)  2)535(356-733) | NA | receive **zidovudine** (300 mg)  daily from 36±38 weeks' gestation until delivery, then in  DITRAME only, for 7 more days.  (-peripartum treatment:  *DITRAME ANRS-049a: 500 mg bid or 600 mg bid  *RETRO-CI: 600 mg bid  -intrapartum treatment:  * DITRAME ANRS-049a: single dose of 500 mg or 600 mg  *RETRO-CI: 300 mg every 3h  -maternal postpartum treatment: for 7 days  * DITRAME ANRS-049a: 500 mg bid or 600 mg bid  *RETRO-CI: none | A maternal short-course zidovudine regimen reduces MTCT of HIV-1 at  age 24 months, despite prolonged breastfeeding (overall Cumulative risks (CR) of mother-to-child transmission (MTCT) were 0.225 in the  zidovudine and 0.302 in the placebo group, a 26% significant reduction). | However, efficacy was observed only  among women with CD4 cell counts > 500/ml. |
| 14 | L. A. Guay P. [31] | randomized, placebo-controlled, double-blind, trial | Kampala,Uganda | Mothers:  1) ziduvudin:308  2) nevirapine:310  Infants:  1) zidovudine:307  2) nevirapine:309 | 1)25(22-28)  2)24(21-27) | 1)65 (21.1%)  2)37 (12%) | NA | 1) 426 (244–638)  2) 461 (291–637) | NA | 1) Two 300 mg **zidovudine** tablets at onset of labour, followed by one 300 mg tablet every 3 h during labour and, for neonates, administration of zidovudine syrup, 4 mg/kg twice daily for 7 days after birth.  2) Single 200 mg **nevirapine** given to the mother at the onset of labour, and a single dose of nevirapine of 2 mg/kg for the neonate at 72 h after birth or at discharge from hospital, whichever occurred first. | Nevirapine lowered the risk of HIV-1 transmission during the first 14–16 weeks of life by nearly 50% in a breastfeeding population. This simple and inexpensive regimen could decrease mother-to-child HIV-1 transmission in less-developed countries. | Nearly all babies (98.8%) were breastfed, with 95.6% still breastfeeding at 14–16 weeks. HIV-1 transmission rates in the zidovudine vs. nevirapine groups were: 10.4% vs. 8.2% at birth (p=0.354), 21.3% vs. 11.9% by 6–8 weeks (p=0.0027), and 25.1% vs. 13.1% by 14–16 weeks (p=0.0006). Nevirapine reduced transmission by 47% (95% CI 20–64) up to 14–16 weeks. Both regimens were well tolerated with similar adverse events. |
| 15 | K. Kintu [32] | randomized, double blind, placebo-controlled trial | Uganda | Infants:  1)vaccine:48  2)placebo:12 | 1) 25 (23, 29)  2) 31 (25, 33) | 1)3  2)1 | Both caesarean and vaginal. | 1)669 (540, 776)  2) 592 (538, 654) | HIV RNA (log10 scale):  1) 3·8 (3·0, 4·2)  2) 4·0 (3·1, 4·2) | **ALVAC-HIV vCP1521** was administered at birth (0-3 days) and at week 4, 8, and 12. | HPTN 027, the first HIV vaccine trial in HIV-exposed infants in Africa, showed that high-quality vaccine trials are feasible in resource-limited settings. The ALVAC-HIV vaccine did not interfere with infants' immune responses to routine childhood vaccines. | Although this was not an efficacy trial, it's notable that no infants contracted HIV after the peripartum period, despite the lack of ARV treatment to prevent transmission through breast milk and a high rate of breastfeeding. |
| 16 | Louise Kuhn [33] | randomized  double-blind placebo-controlled trials | South Africa | Mothers of infants who have Mannose-binding lectin (MBL-2) allele variants: 1)Mothers vitamin A and _-carotene:108  2)Mothers placebo:117 | 1)26.2 ± 4.9  2)26.3± 5 | 1)26(24.1%)  2)35(29.9%) | NA | 1)500±238  2)426±213 | NA | The intervention consisted of a daily supplement containing  **5000 IU retinyl palmitate and 30 mg _-carotene**, which  began between 28 and 32 week of gestation and a 200 000 IU dose  of retinyl palmitate at delivery. An identically looking placebo  was given on the same schedule. | Among infants with Mannose-binding lectin (**MBL**-2) variants, supplementation was associated  with a decreased risk of HIV transmission (odds ratio: 0.37;95%  CI: 0.15, 0.91). | NA |
| 17 | Roger L. Shapiro [34] | randomized controlled trial | Botswana | Mothers:  1.Nevirapine:345  2.Placebo:349 | Median age:  1.27.6  2.27.1 | 1. 15(4.3%)  2.13( 3.7%) | N/A | Median (IQR):  1.356(218-519) (cells/ml)  2.363(250-536) (cells/ml) | Median (IQR):  1.4.3(3.6-4.9) (log10 copies/ml)  2.4.3(3.7-4.9) (log10 copies/ml) | maternal zidovudine from 34 weeks’ gestation through delivery, infant single-dose nevirapine at birth, and infant zidovudine for 1 month. Women were randomized to receive either single-dose nevirapine or placebo during labor. | The strategy's efficacy was similar across groups: HIV infection rates by 1 month were 4.3% in the maternal nevirapine arm and 3.7% in the placebo arm, meeting pre-determined equivalence criteria (95% CI for difference, -2.4% to 3.8%). Nevirapine resistance was detected in 45% of sampled women who received the drug. | *Higher baseline HIV-1 RNA was the only independent predictor of transmission in a multivariate analysis.  * Maternal nevirapine exposure can be avoided without compromising efficacy if adequate antenatal zidovudine is provided.  * Avoiding maternal single-dose nevirapine reduces the risk of developing nevirapine resistance in women. |
| 18 | Ameena E. Goga [35] | Cohort | South Africa | HIV positive mothers: 586  Infants: 653 | N/A | -HIV-exposed infants HIV-positive by 36 weeks: 89 infants  -Infants died by 36 weeks: 67 infants  -Total MTCT transmission (HIV-positive or died by 36 weeks): 156 infants | Vaginal and caesarean section | N/A | 3.7 copies/ml (SD 0.65) (Log viral load) | routine **PMTCT** program included single-dose nevirapine, delayed rupture of  membranes and infant feeding counselling.  the association between 0-12  week feeding practice and HIV-free survival.  -exclusive formula feeding: 309  -exclusive breastfeeding: 313  -mixed breastfeeding: 28 | The study's findings on the effect of breastfeeding on MTCT and HIV-free survival include:  -Low IMR settings: HIV-free survival was poorer with breastfeeding.  -High IMR settings: HIV-free survival was poorer when breastfeeding was stopped early or when infants were mainly/partially breastfed (MBF/ParBF).  -postnatal HIV infection or death by 9 months showed:  -The highest risk was among infants who avoided all breastfeeding in Rietvlei [HR 5.6 (95% CI 1.8, 17)].  -Increased risk for MBF infants in Paarl [HR 4.3 (95% CI 1.2, 16.0)].  -Increased risk for infants who avoided all breastfeeding in Umlazi [HR 4.0 (95% CI 1.2, 13.7)].  Increased risk for MBF infants in Rietvlei [HR 2.7 (95% CI 1.0, 7.2)].  -In higher IMR sites:  -Rietvlei: Avoiding breastfeeding had a higher risk [HR 5.6 (95% CI 1.8, 17)] compared to exclusive breastfeeding (EBF) followed by stopping by 12 weeks [HR 2.8 (95% CI 0.6, 13.1)].  -Umlazi: Avoiding breastfeeding had a higher risk [HR 4.1 (95% CI 1.2, 13.7)] compared to EBF followed by stopping by 12 weeks [HR 1.9 (95% CI 0.7, 5.2)].  -The probability of HIV or death was:  -10.7% ±3% among infants who avoided all breastfeeding in Paarl.  -46% ±11% among infants in Rietvlei who stopped breastfeeding by 12 weeks (p < 0.001). | -95% of the entire study population were ever breastfed from 0 to 12 weeks.  -No infants were exclusively breastfed from 0 to 12 weeks, and 4% were never breastfed from 0 to 16 weeks.  -EBF rates among HIV-positive women dropped from 42% at 3 weeks to 18% at 12 weeks.  -Mixed breastfeeding (mainly PredBF and ParBF) practices were common among HIV-positive women. |
| 19 | Ameena E. Goga[36] | Cross-sectional study | South Africa | Infants:  -2011-12 Survey: 10106  -2012-13 Survey: 9120 | Age of Infants: 4-8 weeks postpartum (specific mean age not provided) | -2011-12 Survey: 2.7% (95% CI: 2.1%-3.2%)  -2012-13 Survey: 2.6% (95% CI: 2.0%-3.2%) | N/A | -2011-12 Survey: Median CD4 count was 359 cells/mm³ (IQR: 240-499)  -2012-13 Survey: Median CD4 count was 372 cells/mm³ (IQR: 264-500) | N/A | Shift from maternal antiretroviral (ARV) treatment or prophylaxis contingent on CD4 cell count to lifelong maternal ARV treatment (**cART**).  Early MTCT measured among mothers receiving any PMTCT intervention, with comparisons between those who received ARVs and those who did not. | **ARV-Exposed** Mothers: Significantly lower unadjusted early MTCT rates compared to **ARV-naive mothers**.  2011-2012: 2.0% (95% CI 1.6-2.5%) vs. 10.2% (95% CI 6.5-13.8%)  2012-2013: 2.0% (95% CI 1.5-2.6%) vs. 9.2% (95% CI 5.6-12.7%)  cART Initiation:  Among mothers who commenced cART during or before the first trimester, early MTCT was 1.2% (95% CI 0.6-1.7%).  Exclusive breastfeeding (EBF) mothers receiving >10 weeks ARV prophylaxis or cART had lower early MTCT (2.2% vs. 12.2%). | N/A |
| 20 | Felicity C. Fitzgerald [37] | Cohort | South Africa | Pregnant women with 9 twin pairs(N= 367 mothers, N= 376 pregnancies)  -ART group: 273  -PMTCT group: 74  -no intervention group: 29 | 27.5 (15 – 44)  -ART group: 27.8 (25–31)  -PMTCT group: 27 (23–32)  -no intervention group: 24.5 (23.5–28.5) | The HIV transmission rate was 5.1%  - HIV-negative infants  (N=206)  - HIV-positive infants  (N=11)  - Infants unknown HIV  status  (N=24) | NVD and caesarean section | 134/µl (IQR 88 – 179)  -ART group: 127 (84–168)  -PMTCT group: 166 (128–267)  -no intervention group: 153 (75–247) | 28 282 copies/ml  -ART group: 30 721 (2 762–50 545)  -PMTCT group: 17 794 (2 762–50 545)  -no intervention group: 40 812 (14 969–91 870) | **ART** (triple therapy with Zidovudine, lamivudine and nevirapine)  the median period on ART before birth was 7.6 weeks. (median gestational week at enrollment: median gestational age 28) | The **length of time on antiretroviral therapy (ART)** before delivery is a significant factor in the transmission of HIV from mother to infant. (All HIV-positive infants were born to mothers who had received less than 8 weeks of ART before delivery.) | Pregnant women were divided into three groups based on their treatment: those who received triple therapy before birth (ART group), those who were referred back to a maternity outpatient unit for PMTCT but did not start ART (PMTCT group), and those who did not receive any intervention before birth or were given AZT prior to birth (No intervention group).  Maternal WHO stage and viral load at first follow-up were additional predictors. Women with WHO stage 3 or 4 had 9.5 times higher transmission risk than those at stage 1. Those with viral load >50 copies/ml at 16 weeks on ART had a 10% transmission rate, while <50 copies/ml had 3% transmission rate. |
| 21 | G. Fatti [38] | Cohort | South Africa | -Age ≤19 years (adolescents):65  -Age 20 - 24 years:247  -age >24 years: 644 | N/A | Vertical HIV transmission rate:  -Adolescent mothers:8.3%  - Age 20 - 24 years: 6.5%  -age >24 years:2.1% | N/A | -Age ≤19 years (adolescents): 383 (296 - 465)  -Age 20 - 24 years: 377.5 (246 - 519)  -age >24 years: 339 (217 - 477) | N/A | *Before April 2010:  -HIV-positive pregnant women with CD4+ ≤200 cells/µL or in WHO stage IV start lifelong **triple ART.**  -Ineligible women get **antenatal** **ZDV** from 28 weeks until delivery and **intrapartum** **sdNVP**.  -Infants get **sdNVP** **after** birth and a **7**-**day** **ZDV** course.  *From April 2010:  -Eligibility expanded to CD4+ ≤350 cells/µL or WHO stages III/IV.  -Ineligible women receive **antenatal** **ZDV** from 14 weeks, **intrapartum** **sdNVP**, and **single-dose tenofovir/emtricitabine post-delivery.**  -Infants get extended NVP based on breastfeeding duration. | younger women had  progressively increased risks of vertical HIV transmission | young women exhibited lower awareness of their HIV status compared to older women. This group also showed slower uptake of antenatal ART, reduced Early Infant Diagnosis (EID) uptake, and a higher Mother-to-Child Transmission (MTCT) rate of HIV, despite having less severe immunosuppression.  Furthermore, adolescents faced increased risks of maternal mortality, first presentation in labor, and stillbirth. |
| 22 | David Etoori [39] | Cohort | southern Swaziland | (Mothers)  N=496 | 26 (IQR: 23, 30) | Of 670 infants, 53.6% received an EID test, 320/359  had a test result recorded and of whom 7 (2.2%) were HIV+. | N/A | 387 cells/μl (IQR: 264, 539) | N/A | (Promote **lifelong ART** for all pregnant and breastfeeding women with HIV, regardless of CD4 count, known as **PMTCTB+** Despite the national standard of care being PMTCTA and the Ministry of Health's recommendations, women were given the option to choose AZT within the PMTCTB+ research study.)  Out of the 496 individuals who began ART, 454 (91.5%) were prescribed Tenofovir+Lamivudine+Efavirenz, while 3 (0.6%) were taking Zidovudine+Lamivudine+Nevirapine, another 3 (0.6%) were on Zidovudine+Lamivudine+Efavirenz, and 36 (7.3%) did not have their ART regimen information available. | In summary, PMTCTB+ demonstrates potential in this context with high rates of maternal viral suppression and minimal transmission to infants. | N/A |
| 23 | Ali Elgalib [40] | Cohort | Oman | (Mothers)N=94  There were 110 pregnancies in 94 women. | 32 ( IQR 25-34 ) | Infant’s HIV status (n = 99)  Negative: 98 (99%)  Positive: 1 (1%)  The rate of  MTCT of HIV was 1% (1/99) | Mode of delivery (n = 104)  Spontaneous vaginal delivery: 49 (47.1%)  Instrumental vaginal delivery: 3 (2.9%)  Caesarean section: 52 (50%)  (Gestational age at delivery, median (IQR) 38 (37–38) wk) | Median CD4 count at baseline: 352 cells/mm3 (IQR, 213-474 cells/mm3); Median CD4 count at or closest to conception: 415 cells/mm3 (IQR, 302-558 cells/mm3) | HIV VL at baseline, median (IQR) 23 182 (5630–97 300) copies/mL  Median VL at or closest to conception, median (IQR) 3300 (20–22 550) copies/mL | 1)**Capacity Building**: -Conducted hands-on training and clinical mentoring for HIV healthcare staff.  -Developed streamlined pathways for HIV diagnosis, ART initiation, switching, and retention in care.  -Newly diagnosed patients received counseling and pre-ART preparation, with follow-ups at 2, 4, 8, and 12 weeks to support adherence and monitor adverse effects.  2)**Case Review**: Analyzing vertical transmissions and near misses.  3)**Counselling**: Experienced counsellors provided initial post-test counselling for HIV-positive pregnant women.  4)**Linkage** **and** **Retention**: Emphasis on care pathways for pregnant women.  On **HAART** at conception (n = 110)  Yes 64 (57.2)  No 46 (41.8)  **Type of ART** during pregnancy (n = 110)  **NNRTI**-based HAART 63 (57.3)  **PI**-based HAART 32 (29.1)  **INSTI**-based HAART 10 (9.1)  None 5 (4.5) | The applied **strategy** resulted in favorable obstetric and virological outcomes for HIV-infected pregnant women from 2016 to 2019, achieving a low rate of mother-to-child transmission (MTCT) of HIV. Oman is now close to finalizing the World Health Organization's validation for the elimination of MTCT of HIV. | Intrapartum ZDV (n = 101)  Yes 88 (87.1)  No 13 (12.9)  ART received by infants (n = 102)  ZDV monotherapy 90 (88.2)  HAART 11 (10.8)  None 1 (1.0)  Preterm labour rate: 21.2%, Low birth weight rate: 15.9%, Antiretroviral coverage during pregnancy: 95.5%, Self-reported adherence during pregnancy: Excellent (74.2%), Good (13.6%), Poor (12.1%), Rate of MTCT of HIV: 1% |
| 24 | Ekouevi Didier K. [41] | Cohort | Côte d'Ivoire | 358 HIV-infected pregnant women eligible for  HAART  PMTCT (No HAART) group: 175  HAART group: 151 | median maternal age was 28 years, (IQR) 25–32 | In utero infected:  PMTCT group - 25/170 (14.3%) 95% CI [11.2-22.9%] (5 stillbirth were excluded. 175-5=170)  HAART group - 3/141 (2.0%)  95% CI [0.7-6.9%] (5 stillbirth and 5 abortions were excluded 151-5-5 = 141) | N/A | Median CD4 cell count was 179 cells/ml (IQR 120–252) | N/A | **PMTCT** group: women received a single intrapartum dose of nevirapine (**NVP**) after a short course of zidovudine (**ZDV**) starting at 36 weeks, or ZDV with lamivudine (**3TC**) starting at 32 weeks until 3 days postpartum.  **HAART** group (MTCT-Plus Program): Included women on HAART before pregnancy and those eligible during pregnancy (WHO stage 2/3 with CD4 <350 cells/ml, or stage 4 with CD4 <200 cells/ml).  They received **HAART** (**ZDV** or stavudine (**d4T**) with **3TC** and **NVP**) during antepartum, labour, and postpartum. | HAART in pregnant women with advanced HIV disease substantially reduced mother-to-child transmission. but was associated with a higher rate of low birth weight. Infant survival rates were similar between HAART and PMTCT groups.  . At 12 months, three paediatric infections (2.3%) occurred  in the HAART group vs. 25 (16.1%) in the PMTCT group (P < 0.001). | The median duration on HAART was 11.7 weeks whereas the median exposure time to short-course antiretroviral drugs (sc-ART) prophylaxis was 4.9 weeks. The most common antiretroviral regimen used was ZDV þ 3TC þ NVP (87%) in the HAART group and ZDV þ sdNVP (54.2%) in the PMTCT group. |
| 25 | François Dabis [42] | open-label intervention Cohort | Abidjan | HIV-infected pregnant women: 1144  Children: 1010  Included in transmission analysis:  -**ZDV: 320**  **-ZDV+NVPsd: 361**  **-ZDV+3TC+NVPsd:329** | Median age:  ZDV+3TC+NVPsd group: 27  ZDV+NVPsd group: 26  ZDV group:25 | Tested HIV positive ≤week 6:  -ZDV: 41(12.8%)  -ZDV+NVPsd: 23(6.4%)  -ZDV+3TC+NVPsd:15(4.6%)  confirmed in utero infection (day 1 or 2):  -ZDV: 3  -ZDV+NVPsd: 10  -ZDV+3TC+NVPsd:7  Probable in utero infection (day 3 or 4):  -ZDV: 2  -ZDV+NVPsd: 4  -ZDV+3TC+NVPsd:3  Intra partum or early postnatal infection:  -ZDV: 17  -ZDV+NVPsd: 8  -ZDV+3TC+NVPsd:3  Timing of peripartum infection unknown:  -ZDV: 19  -ZDV+NVPsd: 1  -ZDV+3TC+NVPsd:2 | Vaginal and caesarean section | Median lymphocyte CD4+ count/mm3: 412 for ZDV+3TC+NVPsd, 370 for ZDV+NVPsd, and 502.5 for ZDV. | Median log10 HIV-1 RNA plasma viral load at enrolment (IQR):  -ZDV+NVPsd:  4.40 (3.76–4.88)  - ZDV+3TC+NVPsd:  4.45(3.94–5.1) | 1) a short-course **peripartum** ZDV regimen combined with NVPsd during labour (**ZDV+NVPsd**) and followed by a very short **neonatal** prophylaxis of **ZDV+NVPsd**; 2) the same regimen with the addition of a maternal short-course of **3TC** (ZDV+3TC+NVPsd)  These strategies were compared to a control regimen, which consisted of administering 300mg ZDV tablets twice daily starting from ≥36 weeks of gestation, a double dose (600mg) at the onset of labor, and a one-week maternal postpartum course of ZDV, without any neonatal prophylaxis. | The six-week transmission probability was 6.5% (95% CI: 3.9–9.1%) with ZDV+NVPsd, representing a 72% reduction compared to ZDV alone (52–88%; p=0.0002, adjusted for maternal CD4, clinical stage, and breastfeeding). With ZDV+3TC+NVPsd, the transmission probability was 4.7% (2.4–7.0%; p=0.34 compared to ZDV+NVPsd). A short-course of ZDV+NVPsd significantly reduces peripartum HIV transmission. | • Maternal tolerance to the three drug regimens was good, with a 0.5% incidence of severe anemia in the first month postpartum. |
| 26 | Coutsoudis [43] | Cohort | South Africa | 549 HIV-1-infected women | Age <30 years: 416 (75.7%)  Age ≥30 years: 131 (23.8%) | At 3 months, the estimated HIV-1 infection rate was 18.8% among 156 never-breastfed children, compared to 21.3% among 393 breastfed children (p=0.5). The Kaplan-Meier estimate showed that infants exclusively breastfed to 3 months had a significantly lower infection rate (14.6%) compared to those who received mixed feeding (24.1%, p=0.03). After adjusting for potential confounders, exclusive breastfeeding was associated with a significantly lower risk of HIV-1 transmission than mixed feeding and had a similar risk to no breastfeeding. | Vaginal and caesarean section | CD4-cell/CD8-cell ratio <0·5: 207 (37.7%)  CD4-cell/CD8-cell ratio ≥0·5: 285 (51.9%) | N/A | Comparison of mother-to-child transmission (MTCT) rates among exclusively **breastfed**, **mixed-fed**, and **formula-fed** (never breastfed) infants. | Exclusive breastfeeding may offer HIV-1-infected women in developing countries a cost-effective and culturally acceptable way to reduce HIV-1 transmission while preserving breastfeeding benefits. | N/A |
| 27 | Carla J. Chibwesha [44] | Cohort | Lusaka, Zambia | HIV-infected pregnant women: 1813 | 29 years (SD ± 5 years) | 3.3%  59 of 1,813 (3.3%; 95% CI 2.5 – 4.2%) infants were HIV-infected at the time of their earliest PCR result (3 – 12 weeks of life). | N/A | Median CD4+ cell count was 231 cells/uL (interquartile range (IQR) 164 – 329) | N/A | impact of **time** between **initiating** highly active antiretroviral  therapy (**HAART**) and **delivery** – duration of antenatal HAART – on perinatal HIV infection. | women who initiated HAART ≤4 weeks before delivery had a 5.2-fold higher risk of HIV transmission compared to those who started HAART at least 13 weeks prior (95% CI: 2.5–11.0).  Maximal effectiveness of prevention of mother-to-child  transmission (PMTCT) programs is achieved by initiating HAART at least 13 weeks prior to  delivery. | - median  duration of antenatal HAART was 13 weeks (IQR 8 – 19)  -Duration of antenatal HAART was the most important predictor of perinatal HIV transmission. |
| 28 | Bulterys [45] | Cohort | Rwanda | HIV-1 seropositive women: 318  HIV-1 seronegative women:309  Children: 184 | N/A | 32 children considered HIV-1-infected – a minimum MCT rate of 32/162=20% [95% confidence interval (CI), 14-26%]. Inclusion of the 22 children who died with indeterminate HIV-1 infection status as probably HIV-1-infected gave us an estimated maximum vertical transmission rate of 54/184=29% (95% CI, 22-36%). | N/A | CD4+:  <0.25:47 (26.5%)  ≥0.25:130( 73.5%) | N/A | Impact of **unprotected sexual intercourse** with increased number of sexual partners during the past 5 years ( before and during pregnancy) on MTCT of HIV-1. | Unprotected sexual intercourse with increased number of partners during the past 5 years was strongly associated with mother-to-child transmission (p<0.001). Maternal CD4/CD8 ratio <0.5 and having more than three sexual partners versus a single partner were significantly associated with increased risk of vertical transmission (OR=2.6, 95% CI: 1.0-6.9 and OR=3.6, 95% CI: 1.1-11.8 respectively). Women with more than one sexual partner during the first trimester of pregnancy were at particularly high risk of transmitting the virus. | N/A |
| 29 | Francesca Bisio [46] | prospective uncontrolled interventional study | Republic of Congo | Mothers: n=415 | Mothers: Median age 23 weeks (IQR 19–28 weeks) | the observed transmission rate among those who completed follow-up was 1.7% (5/290, 95% CI: 0.6%–4.1%). The overall estimated transmission rate in the target population, considering expected transmission among drop-outs, was 10.5%–18.0% (67–115/638). | Vaginal and caesarean section | Median CD4 cell count: 290 cells/mm3 (IQR 167–452 cells/mm3) | N/A | *a maternal **ARV** regimen of **zidovudine/lamivudine/nevirapine**, with adjustments for anemia and drug toxicity, perinatal prophylaxis for mother and child, and postnatal support based on feeding choice. Women with CD4 counts <350 cells/mm³ started ARV immediately, continuing post-delivery and weaning. Those with CD4 counts >350 cells/mm³ began ARV at 28 weeks of gestation, stopping post-delivery or after weaning.  *a single oral dose of nevirapine for mothers at labor onset and for infants at birth. Mothers received zidovudine 300 mg every 3 hours from labor onset until cord clamping, continuing their prenatal ARV regimen. Infants also received zidovudine (4 mg/kg) twice daily from birth to 4 weeks of age. | The study found an observed transmission rate of 1.7% (5/290, 95% CI: 0.6%–4.1%) among participants who completed follow-up. The overall estimated transmission rate, accounting for anticipated vertical transmission among drop-outs, ranged from 10.5% to 18.0% (67–115/638). Comparing this rate to the expected transmission rate without intervention (25%–40%), the program reduced vertical transmissions by approximately 50%. | - Adherence to ARV therapy or prophylaxis was classified as A (ARVs were never stopped), B (ARVs were stopped for fewer than 15 days), C (ARVs were stopped for more than 15 days) or not assessable in 58.6% (243/415), 13.0% (54/415), 26.7% (111/415) and 1.7% (7/415) of women, respectively.  -Factors associated with improved compliance included older age (OR 0.33, 95% CI 0.16–0.66, p=0.002), telephone availability (OR 0.42, 95% CI 0.24–0.72, p=0.002), and occupation (OR 0.57, 95% CI 0.29–1.10, p=0.092). |
| 30 | Lertlakana Bhoopat [47] | Cohort | Thailand | 50 HIV-seropositive pregnant women (seropositive for HIV-1 subtype E)  Short term ZDV:27  Long term ZDV:23 | N/A | 4/27 (14.81%)  All 4 infected neonates were born to mothers who had received the short-term regimen of ZDV prophylaxis. | Vaginal and caesarean section | Short-Term:  Median CD4 cell count: 340 cells/mm³ (20-1240)  Median CD8 cell count: 855 cells/mm³ (520-2033)  Long-Term:  Median CD4 cell count: 400 cells/mm³ (70-1000)  Median CD8 cell count: 960 cells/mm³ (580-1540) | Short-Term:  Plasma viral load (baseline): 4.0 log10 copies/mL (1-5.07)  Long-Term:  Plasma viral load (baseline): 3.4 log10 copies/mL (1-4.62) | Twenty-seven received ‘‘**short**-**term’’** **ZDV**  lasting 14 to 35 days before delivery, whereas the other 23 received  ‘‘**long**-**term’’** **ZDV** lasting 62 to 92 days. (ZDV prophylaxis consisting of 300 mg administered  twice daily, switching to 300 mg administered every 3 hours from the  onset of labor until delivery) | The study found that all infants were tested for HIV-1 up to one year of age. Four neonates tested positive for HIV-1 by PCR on peripheral blood, including one during the neonatal period, all from the short-term prophylaxis group. Placental analysis revealed a higher incidence of HIV proviral DNA expression in the **short-term ZDV** group (67% with positive cells) compared to the **long-term ZDV** group (22%, p < 0.02). This suggests that longer (at least 60 days) ZDV prophylaxis is more effective in reducing HIV expression in the placenta and lowering transmission to neonates. | Placentas were evaluated for HIV infection, showing higher effectiveness of long-term prophylaxis compared to short-term, with fewer positive cells (22% vs. 67%). Neonatal transmission occurred predominantly in the short-term group (4 out of 4 cases). |
| 31 | Dunstan Achwoka [48] | Cross-sectional study | Kenya | Infants:  131,451 (106,452 aged 0–6 months, 20,332 aged 6–12 months, 4,667 aged 12–18 months) | N/A | Overall HIV-Positive Rate: Of the 131,451 PCR tests conducted, 11,439 (8.7%) were HIV-positive.  -HIV-Positive Rates by Age Group:  0–6 months: 6.8%  6–12 months: 14.6%  12–18 months: 27.5%  MTCT Rates Over Time:  -The MTCT rates demonstrated significant trends over time (p < 0.0001). The rates for the 0–6 months and 6–12 months groups decreased over time, whereas the rate for the 12–18 months group increased.  - HIV-Positive Rates by Health System Level:  Primary facilities: 8.77% (95% CI: 8.57, 8.98)  Secondary facilities: 8.50% (95% CI: 8.26, 8.74)  Tertiary facilities: 9.53% (95% CI: 8.80, 10.29)  Although significant differences were observed across health system levels (p = 0.01), there was no consistent trend over time across these levels.  - HIV-Positive Rates by Province:  Central Province: 7.51% (95% CI: 7.10, 7.94) – lowest rate  Coast Province: 10.84% (95% CI: 10.27, 11.43) – highest rate  These provincial differences were significant and remained consistent throughout the study period.  - HIV-Positive Rates by Feeding Options:  0–6 months:  Exclusively breastfed: 6.05% (95% CI: 5.89, 6.22)  Exclusively formula-fed: 5.80% (95% CI: 5.01, 6.67)  Mixed-fed: 9.82% (95% CI: 9.35, 10.32), p < 0.001 (significantly higher)  6–12 months:  Still breastfeeding: 15.79% (95% CI: 15.15, 16.44)  Not breastfeeding: 12.05% (95% CI: 11.22, 12.92), p < 0.001 (significantly higher for breastfeeding)  12–18 months:  Still breastfeeding: 28.17% (95% CI: 26.48, 29.91)  Not breastfeeding: 26.46% (95% CI: 24.20, 28.82) (no significant difference).  - HIV-Positive Rates by ARV Regimen:  Children whose mothers received combination antiretroviral therapy (cART) showed consistently lower HIV-positive rates, with a cumulative rate of 5.6%. | N/A | N/A | N/A | **PMTCT** interventions offered to known HIV-positive mothers.  various ARV regimens:  -34.3% of HIV-positive mothers reported taking combination antiretroviral therapy **(cART**) for their own health.  -20.4% reported taking AZT monotherapy plus single dose nevirapine (**sdNVP**) at the time of delivery.  -4.5% reported taking only **sdNVP** at delivery.  For infants:  -18.5% received daily nevirapine (**NVP**) during the breastfeeding period.  -17.5% received daily **NVP** for six weeks.  -10.4% received **sdNVP** only. | The study evaluated HIV transmission rates among infants born to HIV-positive mothers in Kenya between 2008 and 2013.  -Rates varied by age group, with higher positivity rates in older infants.  - The administration of combination antiretroviral therapy (cART) to mothers was associated with lower HIV-positive rates in their children, highlighting the importance of maternal cART in reducing MTCT rates.  - The data suggests that mixed feeding in infants aged 0–6 months is associated with a significantly higher HIV-positive rate compared to exclusive breastfeeding or formula feeding. In older infants (6–12 months), continued breastfeeding is associated with a higher HIV-positive rate compared to those who are not breastfed. No significant difference was observed in HIV-positive rates between breastfeeding and non-breastfeeding in the 12–18 months age group.  - HIV-positive rates varied across different levels of healthcare facilities and regions, with tertiary facilities and the Coast province showing higher rates. These differences underscore the need for targeted interventions in higher-risk areas. | N/A |
| 32 | Torpey [49] | observational | Zambia | 8237 babies | (0-12 months) | No intervention for mother and infant:  20.9% among infants aged 0–6 weeks  39.3% among infants aged 6–12 months (n = 514)  Intervention for both mother and child:  6.5% among infants aged 0–6 weeks and 15.1% among infants aged 6–12 months  Intervention only for infants:  21.2% among infants aged 0–6 weeks and 28.1% among infants aged 6–12 months  Intervention only for mothers:  8.7% among infants aged 0–6 weeks and 27.2% among infants aged 6–12 months | Home:13.0%  Health facility, c-section:4.9%  Health facility, no c-section:81.7%  Missing:0.2% | N/A | N/A | single-dose nevirapine(**sdNVP**), **zidovudine** + **NVP** or **highly active antiretroviral therapy** for either the child or mother or both | All interventions resulted in significantly lower transmission rate than no intervention. There was no significant difference in the efficacy of these interventions. | Regardless of the intervention, the observed transmission rates were higher among infants aged 6–12 months. |
| 33 | Tookey [50] | Observational | United kingdom-Ireland | 4118 women with 4864 pregnancies | (IQR:30.4(26.6–34.3)) | 1.1% (2003–2007) 0.5 % (2008–2012)  During pregnancy | Vaginal:36.6%  Elective caesarean:39.1%  Emergency caesarean:24.3% | Median(IQR):390 (270–540) cells/mm3 | at baseline  :5200 copies/mL (IQR, 862–22,279 copies/ mL)  at the time of delivery:5083 copies/mL (IQR, 1989–24,900 copies/mL) | Preconception or post conception **lopinavir/ritonavir** (LPV/r) | Low mother to child transmission rate and a decline of transmission rate over time with Use of antepartum antiretroviral therapy (ART) for prevention of mother-to-child transmission (MTCT) and to treat maternal infection, if required, and standard practise in this population; lopinavir/ritonavir (LPV/r) is commonly used. | N/A |
| 34 | Tonwe-gold [51] | observational | Coˆ te d’Ivoire | 250 women:  143 scARV  107 HAART | (IQR:  total :27 (24–31)  HAART: 28 (25–31)  ScSRV: 27 (23–30)) | Overall peripartum transmission:2.2% (95% CI 0.3%–4.2%):  HAART: 1.0% (95% CI 0.0%–3.1%)  ScARV: d 3.1% (95% CI 0.1%–6.1%) in the scARV  Cumulative rate at 12 mo:5.7% (95% CI 2.5%–9.0%)  HAART: 3.3% (95% CI 0.0%–6.9%)  scARV::7.5% (95% CI 2.8%–12.3%) | N/A | Median(IQR): Total:338 (206–488) cells/mm3  HAART: 189 (135–266) cells/mm3  ScARV: 467 (368–602) cells/mm3 | N/A | highly active antiretroviral therapy (**HAART**) as early as 24 wk. of gestation with zidovudine (**ZDV**), lamivudine (**3TC**) and nevirapine (**NVP**) with continuation during labor and postnatally for women with high risk of transmission and **scARV** prophylactic regimens with sc(**ZDV+3TC**) from 32 wk of gestation (until 3 d postpartum) and **sdNVP** in labour or **scZDV** from 28 wk, or **sdNVP** alone, or both **scZDV** and **sdNVP** for women with low risk of transmission.  All infants received **ZDV** syrup for 7 d and **sdNVP** syrup on day 3 | Very low rate of peripartum transmission rate in HAART group and  low rates of postnatal transmission in both HAART and scARV groups in conditions of short breast-feeding exposure | No statistically significant difference according to the infant feeding practice.  low birth weight (,2,500 g) as the only factor associated with acquisition of HIV infection |
| 35 | Pellowski [52] | observational | South Africa | 261 HIV positive women  248 infants for PMTCT outcomes | Maternal:  29.1(5.3) | 0.8% (2/261) after the 6–10week infant PCR test | N/A | During pregnancy (cells/mm3) (IQR):  411 (286–609) | (during pregnancy):  ≥1000 copies/mL :5.8%  ≥40–1000 copies/mL:10.8%  <40 copies/mL:40.4%  No repoet:43.1% | **Maternal** **ART** regimen during pregnancy:  **PMTCT** prophylaxis (zidovudine):16%  **First-line ART** (triple therapy):78%  **Second /third-line ART:** 6%  ARVs during labour only: 0.8%  **Infant** prophylaxis:  **NVP** prophylaxis (87.2%)  **NVP**+**AZT** prophylaxis (12.8) | Lowering transmission rate by PMTCT guidelines. | While South Africa has not yet met MTCT elimination criteria, this study suggests that achieving very high PMTCT coverage and reducing transmission rates in high-prevalence areas may be feasible through enhanced retesting, breastfeeding support, ART adherence, and strengthened data surveillance systems. |
| 36 | Palombi Leonardo [53] | observational | Mozambique, Tanzania, and Malawi | Cohort 1: 809 formula-fed infants  Cohort 2:341 breastfed infants | N/A | At age 1 month:  formula-fed infants:0.8%  breastfed infants:1.2%  At age 6 months:  formula-fed infants:1.8%  breastfed infants:0.8%  The cumulative incidence rate at 6 months of age:  formula-fed infants:2.7%  breastfed infants:2.2% | N/A | N/A | N/A | **DREAM program**:  HAART from the 25^th^ week of gestation to mothers, irrespective of clinical stage, CD4 count, and viral load and post-exposure prophylaxis to infants  Cohort 1: water filters and formula for the first 6 months of lactation  Cohort 2: d HAART for up to 6 months after delivery after being given the option to breastfeed | The DREAM HIV-1 PMTCT protocol was safe and efficacious in reducing transmission in infants of 1 and 6 months of age.  Cumulative transmission at 6 months was 2.7% in formula-fed infants and 2.2% in breastfed infants (P = 0.60), indicating minimal risk difference based on feeding method.  The study confirms that the PMTCT protocol is effective, with results comparable to those in developed countries, and shows no increased postnatal HIV transmission risk associated with breastfeeding when mothers receive HAART. | The mortality rate at 6 months of age:  formula-fed infants:  27 per 1000 person-years  breastfed infants:  28.5 per 1000 person-years.  Both groups showed low anemia and mortality rates, with infant survival rates notably better than those of the general population in Mozambique. |
| 37 | Padua E. [54] | observational | Portugal | 1452 children:  1315 at risk of HIV-1 infection  131 at risk of HIV-2 infection and 6 at risk of dual infection | N/A | 3.4% [95% CI 2.5–4.6%] of HIV-1 and 1.5% (95% CI 0.2–5.4%) of HIV-2 mother–child pairs  A combination of intrapartum, post-partum and in utero transmission. | N/A | N/A | N/A | diverse MTCT prevention protocols and several therapeutic modifications introduced during the period of this study with a shift from zidovudine monotherapy to highly active antiretroviral therapy (**HAART**) | vertical transmission of HIV-1 and the  absence of MTCT prevention during pregnancy were  statistically associated (Fisher’s exact test, P<0.0001) | They observed over time a decreasing rate of  HIV-1 MTCT: 7.0% (95% CI 2.6–14.6%) in 1999; 4.2% (95%  CI 1.6–9.0%) in 2000; 4% (95% CI 1.5–8.4%) in 2001; 3.7%  (95% CI 1.7–7.0%) in 2002; 4% (95% CI 2.0–7.6%) in 2003;  3% (95% CI 1.2–6.0%) in 2004; 0.5% (95% CI 0.0–2.5%) in  2005 (P= 0.007). |
| 38 | Naidoo Keshena [55] | observational | South Africa | 1660 women  819 pre-covid  841 covid period | N/A  (14-41) | Postnatal MTCT rate:  2.1% | N/A | N/A | N/A | **ART** initiation in HIV positive pregnant women | infants born to women on antiretroviral therapy (ART) were 93% less likely to have a positive PCR test than those whose mothers who were not on ART. (OR=0.07, 95% CI 0.031:0.178, p<0.05). | no difference in MTCT between 2019 and 2020 but s a significant increase in new HIV infection after enrolling for antenatal care during the COVID period compared to pre-COVID period (120 vs 62 women, p<0.05) and also a signifcant increase in the HIV prevalence among women who delivered during the COVID period than in the pre-COVID era (43.5% compared to 35.8%, p<0.05)  *The uptake of ART in both the pre-COVID and COVID periods was sub-optimal and did not meet the WHO recommendation of 95%. |
| 39 | Muyunda Brian [56] | Observational | Zambia | 1444 mother-baby pairs  Option A/B:864  Option B+:580 | N/A  (Median age of mothers being 33 (28–38)) | Overall MTCT rate: 5%  option B+: 2.9%  option A/B: 6.93% | Assisted:1.8%  Cesarean:1.3%  Normal vaginal delivery: 96.9% | Mean baseline :467 cells/ml (SD 246.5) | N/A | Option **A**:  **ART** for Life or **AZT** starting at 14 weeks gestation regarding CD4 levels for women and Daily **NVP** from birth until 1 week after breastfeeding cessation or 4-6 weeks if no breastfeeding or mother on triple ART for infants  Option **B**:  **ART** for Life or **d-NVP** and **AZT/3TC** at delivery for 7 days postpartum Triple ARV Prophylaxis at 14 weeks gestation and ending at delivery or 1 week after breastfeeding cessation regarding CD4 levels for women and Daily **NVP** or twice daily **AZT** for 4–6 weeks when replacement feeding. Daily **NVP** for 6 weeks when breast feeding for infants  Option **B+**:  **ART** for Life for women regardless of CD4 levels and Daily **NVP** for 6 weeks for infants | HIV exposed infants of positive mothers on option B+ regimen had a reduced transmission rate compared to the other regimen (P = 0.003) | HEI to women who were married had an increased risk 50% of getting infected compared to those not married [adjusted HR = 1.5; 95% CI = 3.43–6.30; P < 0.001]. Exposed infants whose mothers had assisted delivery had 3 times increased risk of getting infected compared to those born through normal vaginal delivery [Adjusted HR = 3.2; 95% CI = 0.98–10.21; P = 0.050]. |
| 40 | Meda [57] | Open label Cohort | Abidjan &  CoÃ te d'Ivoire & Bobo-Dioulasso & Burkina-faso | Open cohort:  209 women  RCT:  431 | Median age  Open cohort:27 | Open cohort:  19.6% (95% Cl 13.7–25.6%)  at age 15 months  RCT:  21.2% (15.3-27.1) | N/A | Open cohort: 482 cells/µl (IQR: 323– 689/µl) | N/A | oral **zidovudine** (300 mg): one tablet twice a day from 36–38 weeks’ gestation until the beginning of labour, then a single oral dose of 600 mg, then a 7-day postpartum prophylaxis of 600 mg per day  in trial: systematic iron and folate supplements in addition to malaria prophylaxis | **zidovudine** is well accepted and efficacious under routine circumstances | the lower the maternal CD4 cell count, the higher the MTCT rates |
| 41 | Surasak Taneepanichskul [58] | Cohort | Thailand | 50 women | 26.2 ±4.9 | 0  (No HIV-1 genome was detected from the peripheral blood of any newborn within 48 hours of birth) | Normal: 82%  Caesarean section:10%  Vacuum extraction: 4%  Forceps extraction:2%  Breech: 2% | N/A | N/A | **Zidovudine** 250 mg orally twice a day from gestational age 36 weeks until labour.  No ZDV  was given in the intrapartum and newborn period.  No breast feeding was recommended to all HIV positive parturient. | zidovudine treatment in late pregnancy could reduce HIV-1 in utero transmission | No congenital anomaly, birth asphyxia or still birth maternal complications were observed during the postpartum period. |
| 42 | Martinson [59] | Cohort | Coˆte d’Ivoire, Soweto, South Africa and Abidjan | Soweto:  120  Abidjan: 41 | Soweto:26 [IQR]: 22–29  Abidjan: 28 (IQR: 24–31) y | 6 weeks postpartum  Soweto:  First pregnancy: 11.1% (10 of 90 children, 95% CI: 5.5% to 19.5%)  Second pregnancy: 11.1% (12 of 108 children, 95% CI: 5.9% to 18.6%)  Abidjan:  First pregnancy: 13.2% (5 of 38 children, 95% CI: 4.4% to 28.1%)  Second pregnancy: 5.4% (2 of 37 children, 95% CI: 0.6% to 18.2%)  (P = 1.000 and P = 0.449 for Soweto and Abidjan, respectively, in unpaired analysis) | Soweto:  first pregnancy  Normal vaginal: 96 (78.7%)  Cesarean section: 25 (20.5%)  Assisted delivery: 1 (0.8%)  Second pregnancy:  Normal vaginal: 88 (78.6%)  Cesarean section: 23 (20.5%) Assisted delivery: 1 (0.9%)  Abidjan:  first pregnancy  Normal vaginal: 40 (97.6%  Cesarean section: 1 (2.4%)Assisted delivery: 0 (0.0%)  Second pregnancy:  Normal vaginal: 38 (97.4%)  Cesarean section: 1 (2.6%)Assisted delivery: 0 (0.0%) | Median (IQR) CD4 count during second study pregnancy (3106 cells/L)  Soweto: 400 (260–546)  Abidjan: 462 (364–678) | Soweto: N/A  Abidjan:  N/A | **Soweto**: maternal self-reports of taking a tablet of **NVP** at the onset of labor and of not breast-feeding the previous infant during the previous pregnancy and The HIV Network for Prevention Trials (**HIVNET**) 012 **sdNVP** regimen at baseline and 6 weeks postpartum  **Abidjan**:  First pregnancy: a short peripartum course of **ZDV** plus **sdNVP** or **ZDV** plus **3TC** with sdNVP. **Neonates** received **ZDV** syrup (2 mg/kg every 6 hours) for 7 days and sdNVP (2 mg/kg) 3 days after birth.  Second pregnancy:  one of the MTCT regimens evaluated in the Ditrame Plus project | the effectiveness of **sdNVP** when used in successive pregnancies is probably not impaired, possibly because viral resistance selected by prior exposure to sdNVP may wane with time. | In the worst-case scenario (including stillbirths and infant deaths as HIV infected) and using all available HIV test results (unpaired analysis), transmission rates in Soweto and Abidjan were 22.7% (23 of 101 children, 95% CI: 15.0% to 32.2%) and 19.5% (8 of 41 children, 95% CI: 8.8% to 34.9%), respectively, after the initial exposure and 10.7% (11 of 103 children, 95% CI: 5.4% to 18.3%) and 14.6% (6 of 41 children, 95% CI: 5.6% to 29.2%), respectively, after the second exposure to sdNVP (P = 1.000 and P = 0.625 in Soweto and Abidjan, respectively)  *Vertical peripartum transmission  rates did not increase at both study sites despite progression of  HIV disease in the interdelivery period and despite the  assumed presence of resistance mutations selected by prior sdNVP. |
| 43 | Lussiana [60] | Descriptive study(retrospective) | Angola | 104 mothers  107 infants | 29.2 | overall postnatal rate: 13.1% (14/101)  rate in ART group: 1.5% (1/66)  rate in no ART group: 37.1% (13/35)  (odds ratio for HIV transmission: 38.4, 95%CI 4.8–310.7, p = 0.001). | Both caesarean and vaginal.  69.2% vaginally | at first visit in pregnancy:  Mean:  372 (cells/mm3 )  Median: 359 (224–486) | N/A | Sixty-eight women having first visit before or during pregnancy received combination antiretroviral treatment (**ART**) in pregnancy 36 women presented after delivery received no **ART** during pregnancy (In this study, pregnant women with HIV received zidovudine, lamivudine, and nevirapine starting in the third trimester (or earlier if needed). At delivery, women received intravenous zidovudine, and newborns were given oral zidovudine within two hours of birth, continuing for four weeks. Cotrimoxazole was provided to both mothers (if in HIV stage II or higher) and all infants.)  + **zidovudine** to 57 of 107 newborns | Lower transmission and mortality among the women and infants adhering to the **PMTCT** programme (odds ratio for HIV transmission: 38.4, 95%CI 4.8–310.7, p = 0.001). | Overall rate of HIV transmission or death :14.7% (15/102)  rate of HIV transmission or death in worst scenario: 18.7% (20/107) for the entire group, 8.5% (6/71) among infants from mothers with ART and 38.9% (14/36) among infants from mothers with no ART |
| 44 | Luo Ma [61] | Cohort | Kenya | 234 women  299 children | mothers  only have HIV- Kids:  23.21  have HIV+ Kids:  22.9 | 112 of 299 infants were infected.  -Children with DRB concordance with their mothers had a threefold increased risk of perinatal HIV transmission (Odds Ratio: 3.09, 95% CI: 1.64–5.83).  -Children with the DRB3 phenotype were significantly less likely to be perinatally infected (Odds Ratio: 0.304, 95% CI: 0.128–0.723).  -Certain HLA genotypes, such as mothers with the DPB1*55:01 phenotype, were associated with a fivefold increase in the risk of transmitting HIV-1 to their children (Odds Ratio: 5.1, 95% CI: 1.06–24.57). | All vaginally | only have HIV-Kids: 530.8  have HIV+ kids:  563.0 | N/A | to determine the influence of HLA class II genes and  their interactive effect on MTCT of HIV.  All **mothers** and **children** examined in this study were **antiretroviral treatment naïve**.  HLA Genotype Influence:  The study highlighted the significant influence of HLA class II genotypes on the risk of mother-to-child transmission (MTCT) of HIV. Specifically:  -**DRB Concordance**: Increased risk of perinatal HIV transmission was associated with DRB concordance between mother and child. In contrast, DRB discordance and the presence of the DRB3 phenotype in children were protective against MTCT.  -**Maternal DPB1*55:01**: This specific genotype in mothers was linked to a significantly higher risk of MTCT.  -**Children's DPA1*04:01**: This genotype in children was associated with an increased risk of being infected at birth. | The study highlights that HLA genotypes, especially DRB concordance, play a crucial role in the risk of mother-to-child transmission (MTCT) of HIV, emphasizing their importance in preventing perinatal HIV transmission. | -**Biological Markers**: There was no significant difference in CD4+ and CD8+ counts between mothers with HIV-positive and HIV-negative children, suggesting that other genetic factors (such as HLA concordance) played a more critical role in MTCT than traditional markers of HIV progression like CD4+ counts.  -**Duration of Follow-Up**: HIV-negative children were followed up for nearly twice the duration compared to HIV-positive children, though other factors like sex, birth weight, and gestational age were similar between the groups. |
| 45 | Linguissi [62] | Prospective observational | Burkina Faso, West Africa | 378 women | 28.32±0.15 | postnatal  Overall rate: 4.8% (18/378)  Of mothers under HAART:  0.00%  Of mothers under New Prophylactic Protocol (AZT + 3TC + NVP):  6.8% (18/264) | N/A | N/A | N/A | *Prophylactic Antiretroviral Regimen (AZT + 3TC + NVP): Pregnant women received AZT and a single dose of nevirapine before delivery, followed by AZT/3TC during labor and for seven days postpartum. Infants were then given one dose of nevirapine and AZT for 4 weeks after birth.  *HAART (Highly Active Antiretroviral Therapy): A portion of HIV-positive mothers received HAART during pregnancy, which led to a significantly lower MTCT rate compared to the prophylactic regimen, showing no HIV transmission in infants born to mothers under HAART. | The rate of HIV vertical transmission is significantly reduced by **HAART** | PCR is an effective tool to confirm HIV status in pregnant women |
| 46 | V. Leroy [63] | non-randomized open-label prospective Cohort | Abidjan & Coˆte d’Ivoire | Mothers:926  1)ZDV Long-term breastfed:238  2)ZDV+sdNVP Formula-fed:195  3)ZDV+sdNVP shortened breastfed:169  4)ZDV+3TC+sdNVP Formula-fed:126  5)ZDV+3TC+sdNVP shortened breastfed:198 | 1)26(23-30)  2)27(24-31)  3)25(22-30)  4)27(24-30)  5)26(23-31) | 107(11.6%)  CTR (cumulative transmission rate) in groups:  1)22.3%  2)9.4%  3)15.9%  4)5.6%  5)6.8% | NA | 1)487(307-705)  2)377(229-563)  3)358(260-493)  4)398(252-602)  5)419(265-564) | 1)4.05±0.86  2)4.14±0.93  3)4.01±0.84  4)4.35±0.92  5)4.43±0.94 | Mothers received from 32–36 weeks of gestation **scZidovudine (ZDV)+ 2Lamivudine (3TC) +single-dose Nevirapine (sdNVP**) at delivery. Neonates received a **sdNVP+7-day ZDV** prophylaxis. | The two short-course antiretroviral combinations associated to any of the two infant feeding interventions, formula-feeding and shortened breastfeeding, reduce significantly MTCT with long-term benefit until age 18 months and without increasing mortality Each combination had a significantly higher effectiveness than the ZDV long-term breastfed group except for ZDV+sdNVP shortened breastfed children). Effectiveness ranged from 51% to 63%, depending on the regimen and feeding method. Shortened breastfeeding or formula feeding combined with specific antiretroviral treatments significantly reduced MTCT risk and provided long-term benefits up to 18 months without increasing mortality. These results suggest achievable MTCT risk reductions in Africa. | N/A |
| 47 | Seni Kouanda [64] | Cohort | Burkina Faso | 1)Mothers SCART (short-course antiretroviral therapy-monotherapy): 326  -302 received NVP  -24 received AZT.  2)Mothers HAART (Tri therapy):260  - 115 received AZT+3TC+NVP  - 130 received D4T+3TC+NVP  -15 received 2INT+I1IP | **15-24** 1)85(26.3) 2_26(10.3) **25-34**  1)201(62.2) 2)180(68.8) ≥**35**  1)37(11.5) 2)54(20.9) | 1)12/259 (4.6%)  2)0/195 | NA | **<200**  1)29(9.7) 2)123(51.0) **200-500** 1)185(61.7) 2)98(40.7) **≥500**  1)86(28.6) 2)20(8.3) | NA | Assessment of 18 months of maternal **HAART** versus peripartum short-course antiretroviral therapy (**SCART**) regimens for the **PMTCT** of HIV.  1-SCART: NVP or AZT  2-HAART: AZT+3TC+NVP or  D4T+3TC+NVP or  2INT+I1IP | HAART for mothers significantly reduces the risk of infant HIV infection while preserving the breastfeeding option for mothers. | NA |
| 48 | C. Kilewo [65] | open-label, nonrandomized, prospective Cohort study | Tanzania | ***MITRA:**  **mothers/children :398**  *PETRA: 264 | ***MITRA: 26(23-30)**  *PETRA:  26 (23-30) | *MITRA:  19 infants  6 wk: 3.8%  6 mo: 4.9%  *PETRA:  6 wk: 5.4%  6 mo: 11.9% | Both caesarean and vaginal. | *MITRA: 411(269-611)  *PETRA:  459(295-643) | NA | *MITRA study:  - Mothers: received 300 mg of ZDV + 150 mg of 3TC administered twice daily from 36 weeks of gestation, intrapartum, and for 1 week postpartum.  -infants: received an extended treatment: after the first week of ZDV and 3TC, they continued on 3TC alone throughout the breastfeeding period (up to 6 months) and two weeks after stopping breastfeeding.( ZDV (4 mg/kg given twice daily) and 3TC (2 mg/kg given twice daily) from birth to 1 week of age and then with 3TC alone (2 mg/kg given twice daily from weeks 2 to 4 and 4 mg/kg given twice daily after week 4) during breast-feeding (maximum of 6 months) and 2 weeks after stopping breast-feeding.)  *PETRA study:  - Mothers: similar to MITRA  -infants: received ZDV and 3TC for the first week postpartum. (**ZDV** (4 mg/kg given twice daily) and **3TC** (2 mg/kg given twice daily) from birth to 1 week of age). | Prophylactic **3TC** treatment of infants to prevent MTCT of HIV during breast-feeding was well tolerated by the infants and could be a useful strategy to prevent breast milk transmission of HIV when mothers do not need ARV treatment for their own health. | PETRA: did not focus on reducing transmission through breastfeeding.  MITRA: was designed to investigate the impact of ARV treatment during breastfeeding to prevent HIV transmission during this period. |
| 49 | D. Ilboudo J. [66] | Open-label, nonrandomized, prospective Cohort study | Burkina Faso | 1)Mothers:115  2)HBV co-infected:15 | 1)28.1±4.3  2)30(24-37)  NA | 0 | NA | 1)459±206.7  2)381.6±117.8 | Median: 5549.3  Range: 251722 | Combination of **lamivudine** added to **zidovudine** and **nevirapine**  During the pregnancy (HAART) | An antiretroviral therapy that in addition to zidovudine and nevirapine includes lamivudine could block or reduce the vertical transmission in HIV positive pregnant women who are coinfected with HBV. | The rate of vertical transmission of HBV was 21.4% (3/14). |
| 50 | J. Ikechebelu J. [67] | Prospective descriptive study | Nnewi, SouthestNigeria | 1)Mother HAART, baby received PEP (post-exposure prophylaxis), breastfeed:40  2) Mother HAART, baby received PEP, not breastfeed:422  3)Mother HAART, no PEP, not breastfeed:37  4)No HAART, no PEP, breastfeed:112  5)No HAART, received PEP, not breastfeed:47  6)No HAART, no PEP, not breastfeed:19 | NA | 1)5(12.5%)  2)12(2.8%)  3)3(8.15%)  4)42(37.5%)  5)5(10.6%)  6)4(21.1%) | NA | NA | NA | **PMTCT** **programme** (Mothers on HAART, babies receiving ARV, not breastfeeding)  HAART starting from 14 weeks, usually with zidovudine (ZDV) 300mg BD, lamivudine (3TC) 150mg BD and nevirapine (NVP) 200mg BD. HIV positive women on HAART who become pregnant were continued on HAART through the pregnancy. Treatment was continued during labour and Postnatally. | The use of HAART in PMTCT programme in the under resourced areas can achieve similar success rates to that in the industrialized countries. Breastfeeding reduces the efficacy achieved by the use of ARV drugs. | All infants received NVP syrup 2 mg/kg and ZDV syrup 4 mg/kg |
| 51 | R. M. Hoffman V. [68] | Prospective descriptive study | Johannesburg, South Africa | 1)mothers starting HAART during pregnancy:968  2)becoming pregnant on HAART:174 | 30.2 ± 5 | 1) 5.7%  2) 0.7% | NA | 1) 155.5 cells/mm3  2) 187.7 cells/mm3 | NA | highly active antiretroviral  therapy (**HAART)**  1) women starting HAART during pregnancy  2) women becoming pregnant on HAART | Women who became pregnant on HAART had significantly lower MTCT rates than women who initiated HAART during pregnancy (0.7% versus 5.7%; *p*=0.01; 95% CI 0.02-3.8 and 4.2-7.7, respectively). Moreover, transmission decreased with longer duration of HAART: 9.3% (14/151) with less than 4 weeks of HAART during pregnancy, 5.5% (23/422) with 4-16 weeks of HAART, and 3.5% with >16 to 32 weeks. Of note, there were no transmissions among women who were on HAART for more than 32 weeks prior to delivery. | *MTCT rates in women who receive HAART during pregnancy:  - single dose nevirapine prophylaxis: 7.9%  - <4 weeks HAART during pregnancy: 9.3%  - 4-16 weeks of HAART during pregnancy: 5.5%  - >16 to 32 weeks of HAART during pregnancy: 3.5%  * Every additional week of therapy  reducing the odds of transmission by 7% |
